# Supplementary material for: Antioxidant, Cytotoxic, and Antimicrobial Activities of Glycyrrhiza glabra L., Paeonia lactiflora Pall., and Eriobotrya japonica (Thunb.) Lindl. Extracts
Source: Medicines (Basel). 2019 Mar 30;6(2):43. doi: 10.3390/medicines6020043 (PMC6631708; doi:10.3390/medicines6020043)
Supplement: Supplementary file 1 [file medicines-06-00043-s001.pdf]

Supplementary Materials: Antioxidant, Cytotoxic, and Antimicrobial Activities of *Glycyrrhiza glabra* L., *Paeonia lactiflora* Pall., and *Eriobotrya japonica* (Thunb.) Lindl. Extracts

Jun-Xian Zhou, Markus Santhosh Braun, Pille Wetterauer, Bernhard Wetterauer and Michael Wink

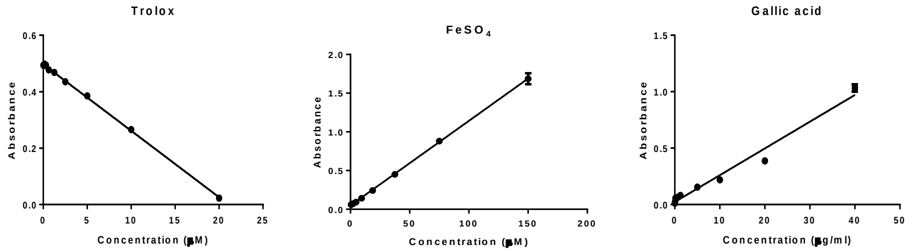

Figure S1. The standard curves in the TEAC, FRAP and Folin-Ciocateu assays shown as absorption vs. concentration. Results are expressed as the mean ± SD from at least three independent experiments.

Table S1. Secondary metabolites in *Glycyrrhiza glabra.*

Part	Class	Plant Secondary Metabolites	References
Root	Triterpenes	Glycyrrhizic acid	1-6
		Glabric acid	7
		Liquoric acid	8
		Betulinic acid	9
		18α-Glycyrrhetinic acid<br>18β-Glycyrrhetinic acid	2,3,5,10-12
		Ammonium glycyrrhinate	10
		Isoglabrolide	13
		21α-Hydroxyisoglabrolide	13
		Glabrolide	13
		11-Deoxyglabrolide	13
		Deoxyglabrolide	13
		Glycyrrhetol	13
		24-Hydroxyliquiritic acid	13
		Liquiridiolic acid	13
		28-Hydroxygiycyrrhetinic acid	13
		18α-Hydroxyglycyrrhetinic acid	13
		Olean-11,13(18)-dien-3β-ol-30-oic acid and 3β-acetoxy-30-methyl ester	13
		Liquiritic acid	13
		Olean-12-en-3β-ol-30-oic acid	13
		24-Hydroxyglycyrrhetinic acid	13
		11-Deoxyglycyrrhetinic acid	5,13
		24-Hydroxy-11-deoxyglycyirhetinic acid	13
		11-Desoxoglycyrrhetic acid acetate methyl ester	14
		24-Acetoxy-11-desoxoglycyrrhetic acid acetate methyl ester	14
		11-Desoxoglabrolide acetate	14
		Glabrolide acetate	14
		3β-Acetyl-18β-hydroxy-11-keto-olean-12-en-30-oic acid, 30,18β-lactone	14
		22β-Acethylglabric acid	15
		Glycyrrolide I	15
		3β-Acetyoxyglybrolide	15
		3β-Acetyoxy-11-deoxoglybrolide	15

		3-Acetyoxy-11-deoxo-glycyrrhetinic acid	15
		Methyl-24-hydroxy-11-deoxo-glycyrrhetate	15
		Triphyllic acid	15
		Isomacedonic acid	15
		Licorice saponin A3	5,16
		Licorice saponin C2	5
		Licorice saponin G2	5,16,17
		Licorice saponin J2	5
		Glycyrrhizin isomer	5
		22-Acetoxyglycyrrhizin	5
		24-Hydroxylicorice saponin A3	16
		22β-Acetoxyl-glycyrrhizin	16
		Licorice saponin M3	16
		Licorice saponin N4	16
		Licorice saponin O4	16
		18α-Licorice saponin G2	18
		Macedonoside A	18
		29-Hydroxy-glycyrrhizin	18
		Licorice saponin K2/H2	17
		Licorice saponin B2	17
		β-Sitosterol	19
		11-Deoxoglycyrrhizin	6
		24-Hydroxyglycyrrhizin	6
		Glucoglycyrrhizin	6
		Araboglycyrrhizin	6
		Apioglycyrrhizin	6
		Glycyrrhetinic acid-monoglucuronide	6
		11-Deoxo-11,13-glycyrrhizindiene	6
	Monodesmosidic saponins	30-Hydroxyglycyrrhizin	18
		Glycyrrhizin-20-methanoate	6
		24-Hydroxyglucoglycyrrhizin	6
		Rhaoglycyrrhizin	6
		11-Deoxorhaoglycyrrhizin	6
		Rhaoglucoglycyrrhizin	6
		Rhaogalactoglycyrrhizin	6
		11-Deoxo-20α-glycyrrhizin	6
		20α-Galacturonoylglycyrrhizin	6
		20α-Rhaoglycyrrhizin	6
	Sesquiterpenes	*β*-Caryophyllene	20
		β-Caryophyllene oxide	20
		Himachalene epoxide	20
		(1*R*,3*E*,7*E*,11*R*)-1,5,5,8-tetramethyl-12-oxabicyclo[9.1.0]dodeca-3,7-diene	21
		Caryophyllene oxide	21
	Monoterpenes	Camphor	22
		(*Z*)-Pinene hydrate	20
		Terpinen-4-ol	20
	Isoflavones	Formononetin	6,23-25
		Prunetin	27
		Glabrone	28,29
		Glyzarin	30
		Licoisoflavone A	31
		Glycyrrhisoflavanone	32
		Glycyrrhisoflavone	32
		Glycyrrhisoflavanone diacetate	32
		Glycyrrhisoflavanone tricetate	32
		Glycyrrhisoflavone tetraacetate	32
		Licoisoflvone A	32
		Glisoflavone	33

		6,8-Di-(dimethylallyi)-genistein	34
		3',6-Di-(dimethylallyl)-genistein	34
		Licoisoflavone B	34
		Licoisoflavanone	34
		Shinpterocarpin	29,35
		Glabrone	15
		Glyzaglabrin	36
		Glabroisoflavanone A	37
		Glabroisoflavanone B	37
		Genistein	6
		Daidzin	6
		Genistin	6
		Daidzein	6
		Glycitein	6
		Coumestrol	6
		O-Methylshinpterocarpin	29
		Gancaonin L	29
		7,8-Dihydroxy-4'-methoxy-6-prenylisoflavanone	29
		2',3-Dihydroxy-4'-methoxy-3'',3''-dimethylpyrano [2'',3'':7,8] isoflavanone	29
		Erybacin B	38
		Parvisoflavones A	38
	2-Methylisoflavones	7-Atetoxy-2-methylisoflavone	39
		7-Methoxy-2-methylisoflavone	39
		7-Hydroxy-2-methylisoflavone	39
	Isoprenoid-substituted isoflavones	Kanzonol T	34
	Isoflavone glycosides	Ononin	6,40
	Isoflavans	Glabridin	5,17,24,29,35, 41-44
		4'-*O*-Methylglabridin	24,29,35,42,45
		Hispaglabridin A	17,24,29,35,42
		Hispaglabridin B	24,29,42
		Phaseollinisoflavan	24
		3'-Methyoxy-glabridin	15
		8-Prenyl-phaseollinisoflavan	42
		3'-Hydroxy-4-*O*-methylglabridin	29,42
		5'-Formyl glabridin	29
		4''-Hydroxyglabridin	29
		(3*R*)-Vestitol	29
		8-Hydroxymethyl-8-methyl-3,4-dihydro-2*H*,8*H*-Pyrano[2,3-*f*]-chromon-3-ol	29
	Hydroxyisoflavans	Licoricidin	46
	Dipyranoisoflavans	Glyinflanin K	47
	Prenylated isoflavans	Kanzonol R	48
	Diprenylated isoflavans	Kanzonol X	29,35
		(3R)-2',3',7-Trihydroxy-4'-methoxyisoflavan	29
	Isoflavene	Glabrene	5,24,28,29,35
	Isoflavene derivatives	3,4-Didehydroglabridin	37
	Pterocarpans	Medicarpin	47
		Iicoagrocarpin	29
		Kanzonol F	5
	Chalcones and chalcone derivatives	Licochalcone A	34,49,50
		Licochalcone B	5,49
		Licuroside	5,51

		Neoisoliquiritin	51
		Echinatin	29,51
		Isoliquiritin	35,40
		Glyinflanin G	35
		Kanzonol Y	5,17,29,35,47
		Rhamnoisoliquiritin	15
		3, 3'-Di-γ,γ-dimethylallyl-2'-4,4'-trihydroxychalcone	52
		[6",6"-Dimethylpyrano(2",3":4,5)]-3'-γ,γ-dimethylallyl-2',3,4'-trihydroxychalcone	29,52
		Licuraside (neolicuroside)	5,47
		2,3,4-Trihydroxy-4'-methoxychalcone	53
		2,4,4'-Trihydroxychalcone	53
		3,3',4,4'-Tetrahydroxy-2'-methoxy-5-prenylchalcone	29
		2,3',4,4'-Tetrahydroxy-3,5'-diprenylchalcone	29
		2,3',4,4',α-Pentahydroxy-3,5'-diprenyl-dihydrochalcone	29
		2,3',4,4',α-Pentahydroxy-3-prenyl-dihydrochalcone	29
		Morachalcone A	29
		Isoliquiritigenin	5,54-57
		Licorice glycoside A	5,
		2,4,4'-Trimethoxychalcone	17
		Glyinflanin A	17
		Licoflavone B	17
		Glycyrdione B	17
		Glyinflanin D or G	17
		Licochalcone D	17
	Retrochalcones	Lichocalcone B (3, 4, 4'-trihydroxy-2-methoxychalcone)	29
	Hydroxydihydrochalcones	1-(2,4-Dihydroxyphenyl)-2-hydroxy-3-(4'-hydroxyphenyl)-1-propanone	53
		1-(2,4-Dihydroxyphenyl)-3-hydroxy-3-(4'-hydroxyphenyl)-1-propanone	53
	Hydroxydihydrochalcone glucosides	1-(2,4-Dihydroxyphenyl)-3-hydroxy-3-(4'-hydroxyphenyl-4'-*O*-β-D-glucopyranoside)-1-propanone	53
		1-(2,4-Dihydroxyphenyl-4-*O*-β-D-glucopyranoside)-3-hydroxy-3-(4'-hydroxyphenyl)-1-propanone	53
	Flavonosides	Liquiritoside	58
	Flavonoids	Pinocembrin	27
		Glyasperin M	34
		Euchrenone a_5	29,47
		Rhamnoliquirilin	59
	Flavonoid dimer	Glabridin + sophoracoumestan A	17
		Glabridin + sophoracoumestan A	17
		Glabrone + glabridin	17
		Glabrone + glabridin	17
	Isoprenoid-substituted flavonoids	Gancaonin F	60
		Gancaonin G	34,60
		Gancaonin H	34,60
		Gancaonin I	60
	2-Arylbenzofuran flavonoids	Licocoumarone	33,61
		Kanzonol U	29,35
		Kanzonol V	35
		Glabrocoumarone A	52
		Glabrocoumarone B	52
		ω-Hydroxymoracin N	17
	2-Arylbenzofuran dimer	Lespedezol B3	17
	Flavonols	Licoflavonol	62
		Kumatakenin	62
		Licoricone	62
		Isolicoflavonol	32

		Astragulin	15
		Kumatakenin	63
	Flavones	4',7-Dihydroxyflavone	32
		Kaempferol 3-*O*-methyl ether	33
		Saponaretin (isovitexin)	51
		Glabranin	51
		Genkwanin	15,51
		Quercetin-3,3'-dimethylether	15
		Quercetin-3-glucobioside	15
		5,8-Dihydroxy-flavone-7-*O*-beta-D-glucuronide (glychionide A)	64
		5-Hydroxy-8-methoxyl-flavone-7-*O*-beta-D-glucuronide (glychionide B)	64
		Licoflavanone A	29
		Isoviolanthin	5
		Luteolin	65
		Apigenin	65
	Flavanones	Glabrol	5,24,29,35,41,52
		Liquiritin	5,40,66, 67
		3',6- and 3',8-Diprenylated dalbergioidin (2',4',5,7-tetrahydroxy-isoflavanone)	34
		Isoliquiritoside	15
		3-Hydroxygrabrol	52
		Shinflavanone	29,52
		[6'',6''-Dimethylpyrano(2'',3'':7,8)]-[6''',6'''-dimethylpyrano(2''',3''':4',3')]-flavanone (xambioona)	29,52
		(2*R*,3*R*)-3,4',7-Trihydroxy-3'-prenylflavanone	29
		Liquiritigenin	3,4,56,57,68
		Choerospondin	5
		Licorice glycoside D2/D1	5
		5,7-Dihydroxyflavanone	5
		3-Hydroxyglabrol	5
		Rhamnoliquiritin	5
		Pinocembrin	67
	3-Hydroxypyranoflavanones	Kanzonol Z	47
	Flavonoid glycosides	Neoliquiritin	51
		Violanthin	40
		Isoschaftoside	40
		Schaftoside	17,40
		Liquiritin apioside	5
	Phenylpropanolds	Sinapinic acid	65,68
		Ferulic acid	23,65,68
		Phenylpropionic acid	69
		Eugenol	20,22
		Caffeic acid	65
	Coumarins	Herniarin	68
		Umbelliferon	68
		Liqcoumarin	70
		Glabrocoumarin	29,37
		Glycocoumarin	63
		Licofuranocoumarin	63
	3-Arylcoumarins	Glycyrin	71
		Glycycoumarin	32,61
		Licoarylcoumarin	33
		Licopyranocoumarin	33
	Pyrano-3-arylcoumarin	Kanzonol W	29,35
	Coumestan	Glycyrol	9,60,62

	derivatives	5-*O*-Methylglycyrol	9
		Isoglycyrol	9,60
	Phenolic compounds	Mulberrofuran K	17
	Diarylheptanoid	Kuwanon V	17
	Benzophenone	Cudranone	17
	Diels-Alder types adducts	Guangsangon F	17
	Carboxylic acids	Benzoic acid	69
		p-Ethoxybenzoic acid	69
		Gentisic acid	65
		p-Coumaric acid	65
	Phenols (essential oil) Acohols and ethers (essential oil)	Guaiacol (*O*-methoxyphenol)	22,69
		Phenol	69
		p-Methoxyphenol	69
		2,4-Dihydroxyacetophenone	69
		Ethylphenol	69
		(*O*-)Cresol	69
		2-Methalyl phenol	22
		3-Methyl-6-propyl phenol	22
		2-Methyl-5-isopropyl phenol	22
		p-Vinyl-guaiacol	20
		Hexanol	22,69
		2-Phenylethanol	69
		4-Methyl-1-isopropyl-3-cyclohexen-l-ol (α-terpineol)	20,22,69
		2,3-Butanediol	69
		1,2-Heptanediol	69
		Linalool	69
		Lavandulol	69
		Cymenol	69
		Benzyl alcohol	69
		Phenylethyl alcohol	22,69
		Dimethylphenethyl alcohol	69
		Linalyl oxide	69
		4-Propenylanisol	69
		n-Pentanol	22
		2-Methyl-6-methylen-7-octen-2-ol	22
		Cumic alcohol	22
		1-Pentadecanol	22
		Lavandulol<tetrahydro->	20
		(*E*)-Linalool oxide	20
		p-Cymen-8-ol	20
		Methyl chavicol	20
		α-Cadinol	21
	Aldehydes (essential oil)	Tiglaldehyde (tentative)	69
		Benzaldehyde	69
		2-Hexanal	22
		Myrtenal	22
		Phenylacetaldehyde	22
		7-Methoxy-3,7-dimethyl-octanal	22
		2-Hydroxy-4-methyl benzaldehyde	22
		Cumin aldehyde	20
		(*E*)-Cinnamaldehyde	20
		(4*E*)-Decenal	20
		Benzaldehyde	21
		Caproaldehyde	21
		Nonanal	21
	Ketones and	1-Butanol-2-one (tentative)	69

	hydroxy ketones (essential oil)	1-Butanol-3-one	69
		Acetoin (tentative)	69
		2-Hydroxy-3-methyl-2-cyclopenten-l-one	69
		4-Hydroxy-4-methyl-2-pentanone	69
		Acetol	69
		Methyl ethyl ketone	69
		Fenchone	69
		Thujone	69
		6-Methyl-5-hepten-2-one	22
		3-Methyl-3-hepten-2-one	22
		3,5-Octadien-2-one	22
		6-Methyl 3,5-heptadien-2-one	22
		Acetonphenoone	22
		n-Methyl-2-pyrrolidone	22
		2-Methyl-5-isopropyl-2-cyclohexen-1-one	22
		Pseudoionone	22
		5-Pentylpyran-2-one	22
		2-Methyl-3-decen-5-one	22
		Carvone	20
		Piperitone	20
		Geranylacetone	21
		6,10,14-Trimethyl-2-pentadecanone	21
	Esters (essential oil)	Ethyl palmitate	69
		Ethyl linoleate	69
		Ethyl linolenate	69
		Ethyl phenylacetate	69
		Butyl phthalate	69
		Butyric anhydride (tentative)	69
		Butyrolactone	69
		γ-Hexalactone	69
		γ-Heptalactone	69
		γ-Nonalactone	20,22,69
		γ-Octalactone	69
		Propyl *p*-hydroxybenzoate	69
		Methyl hexanoate	22
		Hexyl formate	22
		Isobutyl adipate	22
		Methyl hexadecanoate	22
		Hexadecyl acetate	22
		Hexadecanoic acid, ethyl ester	72
		Linoleic acid ethyl ester	72
		E-8-Methyl-9-tetradecen-1-ol acetate	72
		9,12,15-Octadecatrienoic acid,ethyl ester, (Z,Z,Z)-	72
		γ-Lactones (C6–C14)	72
		4-Methyl-γ-lactones	72
		4-Ethyl-γ-lactones	72
		Methyl 11-cyclopentylundecanoate	21
	Hydrocarbons (essential oil)	*p*-Cymene	69
		4-Propenyltoluene	69
		6-Methyl-3-undecene	22
		5-Methyl-2-undecane	22
		9-Methyl-3-undecene	22
		1-Docosene	72
		1-Hexadecene	72
		1-Octadecene	72
		Undecane,4-cyclohexyl-	72
		5-Acetoxypentadecane	72
		4-Methyloctane	21

		2,3-Dimethyl-dodecane	21
	Aromatic compounds (essential oil)	Carvacrol	69
		Thymol	69
		Estragole (methyl cavicol)	22
		Anethole	22
		Styrene	21
		2-Pentylfuran	21
		2,2'-Methylenebis(6-tert-butyl-4-methylphenol)	21
		(*E*)-Anethole	20
		Thymol	20
		Carvacrol	20
	Furan derivatives (essential oil)	Furfural	22,69
		Furfuryl alcohol	69
		5-Methyl-3-hydrofuran-2-one	69
		2-Methyl-2-tetrahydrofuran-3-one	69
		2-Acetylfuran	69
		5-Methylfurfural	20,69
		Dihydrobenzofuran	69
		2-Acetyl- 5-methylfuran	69
		1-(2-Furyl)-2-propan one (tentative)	69
		Furyl ethyl ketone	69
		1-(5-Methyl-2-furyl) l,2-Propanedione	69
		Furfuryl formate	69
		Furfuryl acetate	69
		Furfuryl propionate	69
		Furfuryl butyrate	69
		2,2-Difurylme thane	69
		2,2-Difurylethane (tentative)	69
		2,2-Difurylethylene (tentative)	69
		Difurfuryl ether	69
		1-Furfuryl-2-formylpyrrole	69
		1-Furfuryl-2-acetylpyrrole	69
		2,4-Difurfurylfuran (tentative)	69
		2-3-Dihydro-4-methyl furan	22
		2-Pentylfuran	22
		2,3-Dihydro benzofuran	22
		Dihydro-5,5-dimethyl-2(3*H*)-furanone	22
	Furanones	Coffee furanone (2-methyl-tetrahydrofuran-3-one)	51
	Heterocycle compounds	2-Acetylpyrrole	69
		1-Methyl-2-formylpyrrole	69
		2-Formyl-5-methylpyrrole	69
		Pyrazole (tentative)	69
		2,6-Dimethylpyrazine	69
		2-Ethyl-6-methylpyrazine	69
		Trimethylpyrazine	69
		Maltol	69
		Furylmethylketone	69
		2-Methyltetrahydrofuran-3-one	69
		2-acetylpyrrole	69
		Indole	22,69
	Toluenes	2-Ethyl-1,4-dimethyl benzene	22
	Miscellaneous compounds	1-Methoxy-4-isopropyl cyclohexane	22
		3-Methoxy-2-methyl propane	22
		O-Tolunitrile	22
		1-Methoxy-4-isopropyl benzene	22
		8,8-Dimethyl-3,4-dihydro-2*H*,8*H*-pyrano[2,3-*f*]-chromon-3-ol	29
		Methyl eugenol	20
	Sesquiterpenes	Caryophyllene	72

Leaf		D-cadinene	21
		Caryophyllene oxide	21,72
		Germacrene D	72
		Humulene- (VI)	72
	Monoterpenes	D-limonene	72
		Calacone	21
	Prenylated dihydrostilbenes	α,α′-Dihydro-3,5,4′-trihydroxy-4,5′-diisopentenylstilbene	74
		α,α′-Dihydro-3,5,3′,4′-tetrahydroxy-4,5′-diisopentenylstilbene	74
		α,α′-Dihydro-3,5,4′-trihydroxy-5′-isopentenylstilbene	74
		α,α′-Dihydro-3,5,3′-trihydroxy-4′-methoxy-5′-isopentenylstilbene	74
		α,α′-Dihydro-3,5,3′,4′-tetrahydroxy-5′-isopentenyl stilbene	74
		Dihydro-3,5-dihydroxy-4′-acetoxy-5′-isopentenylstilbene	75
		Dihydro-3,3′,4′-trihydroxy-5-o-isopentenyl-6-isopentenylstilbene	75
		Dihydro-3,5,3′-trihydroxy-4′-methoxystilbene	75
		Dihydro-3,3′-dihydroxy-5beta-d-o-glucopyranosyloxy-4′-methoxystilbene	75
	Isoflavones	Formononetin	4,25
		Wighteone	74,76
		Genistein	76
		Lupiwighteone	75,76
		Prunetin	76
	Flavonones	Licoflavanone	25,76
		Pinocembrin	25,67,74,76
		Naringenin	75
		Liquiritin	67
	Flavonoids	Glabranin	67,74
		Licoflavone	74
		Glabranin isomer	75
		Pinocembrin 7-*O*-glucoside	75
		Astragalin	75
		Vicenin II	75
		Pinocembrin-7-*O*-β-D-glucopyranoside	67
		Pinobanksin	67
		Galangin	67
	Flavonoid glycosides	Isoquercitrin	25
	Cyclic polyols	Pinitol	75
	Acohols	Benzyl alcohol	21
		Nerolidol	21
		α-Cadinol	21
		Phytol	21
	Aldehydes	Tetradecanal	72
		Nonanal	72
		3-Methyl-2-butenal	21
		Trans-2-hexenal	21
		Benzaldehyde	21
		2-Phenylethanal	21
	Ketones	6-Prenylnaringenin	76
		2-Undecanone, 6,10-dimethyl-	72
		2-Pentadecanone, 6,10,14-trimethyl-	72
		7,8-Epoxy-2-ionone	72
		Benzylacetone	21
		1- Divinyl-ethanone	21
		Beta-damarone	21
		Geranylacetone	21
		β-Ionone	21
		4-[2,2,6-Trimethyl-7-oxabicyclo[4.1.0]hept-1-yl]-3-buten-2-one	21
		6,10,14-Trimethyl-2-pentadecanone	21

	Esters	Dibutyl phthalate	72
		Dihydroactinidiolide	21
		Benzyl benzoate	21
		1,6-2 Benzoate	21
	Aromatic compounds	Benzocyclobutene	21
	Phenols	2,2'-Methylenebis(6-tert-butyl-4-methylphenol)	21
	Acids	Pheracetic acid, 2,5α,α-tetramethyl-	72
		Phthalic acid	21
	Alkanes	Pentane, 2-methyl-	72
		Pentane, 3-methyl-	72
		Pentane, 2,2-dimethyl-	72
		Cyclopentane, methyl-	72
		Pentane, 3,3-dimethyl-	72
		Hexane, 2-methyl-	72
		Pentane, 2,3-dimethyl-	72
		Haxane, 3-methyl-	72
		Pentane, 3-ethyl-	72
		Cyclopentane, 1,2-dimethyl-	72
		Pentane, 2,2,4-trimethyl-	72
		Cyclohexane, methyl-	72
		Hexane, 2,4-dimethyl-	72
		Hexane, 3,3-dimethyl-	72
		Cyclopentane, 1,2,3-trimethyl-	72
		Pentane, 2,3,3-trimethyl-	72
		Hexane, 2,3-dimethyl-	72
		Heptane, 2-methyl-	72
		Hexane, 3,3,4-trimethyl-	72
		Hexane, 3,4-dimethyl-	72
		Heptane, 3-methyl-	72
		Hexane, 3-ethyl-	72
		Cyclphexane, 1,3-dimethyl-, trans-	72
		Hexane, 2,2,4-trimethyl-	72
		Cyclohexane, 1,1-dimethyl-	72
		Cyclopentane, 1-ethyl-3-methyl-	72
		Cyclopentane, 1-ethyl-2-methyl-	72
		Cyclopentane, 1-ethyl-1-methyl-	72
		Cyclopentane, 1,2-dimethyl-, trans-	72
		Octane, 2-chloro-	72
		Cyclohexane, 1,4-dimethyl-, cis-	72
		Cyclopentane, (1-methylethyl-)	72
		Hexane, 2,3,5-trimethyl-	72
		Hexane, 2,2,5-trimethyl-	72
		Heptane, 2,4-dimethyl	72
		Pentane, 3-methyl-3-ethyl-	72
		Heptane, 2,6-dimethyl	72
		Cyclohexane, ethyl-	72
		Heptane, 2,5-dimethyl-	72
		Hexadecane, 2,6,10,14-tetramethyl	72
		Nonadecane, 9-methyl-	72
		Hexadecane, 2,6,10,14-tetramethyl-	72
	Hydrocarbons	4-Methyloctane	21
		Ethylbenzene	72
		p-Xylene	72
	Miscellaneous compounds	Naphthalene, 1,2,3,4-tetrahydro-1,6-dimethyl-4-(1-methylethyl-)-(1s-*cis*)-	72
		N-Phenyl-*1*-naphthylamine	72
		Benzene, 1-methoxy-4-(2-propenyl)-	72
		Benzene, 1-methoxy-4-(1-propenyl)-	72

Seed	Flavonoid glycosides	Isoquercitrin	25
Stem	Flavonones	Pinocembrin	25
		Licoflavanone	25
Stolon	Flavanones	3-Hydroxyglabrol	35
Root and stolon	Flavanones	3-Hydroxyglabrol	35
	Triterpenes	Glycyrrhizic acid	77,78
	Isoflavones	Glisoflavone	33
		Formononetin	79
	Isoflavans	Glabridin	79
		Hispaglabridin B	79
		4′-*O*-Methylglabridin	79
	Chalcones	Isoliquritigenin	77-79
		Kanzonol Y	35
	Chalcone derivatives	1,2-Dihydroparatocarpin A	79
	Flavones	Kaempferol 3-*O*-methyl ether	33
	3-Arylcoumarins	Licoarylcoumarin	33
		Licopyranocoumarin	33
	2-Arylcoumarins	Licocoumarone	33
	Neolignan lipid esters	Mixture of neolignan lipid esters	79
	Phenolic compounds	Hemileiocarpin	79
		Paratocarpin B	79
Root and rhizome	Triterpenes	Glycyrrhetinic acid	80,81
		Glycyrrhizic acid	81,82
		Uralsaponin B	82
	Isoflavones	Glabrene	83
		Calycosin	54
		Shinpterocarpin	45
		Derrone	45
		Lupiwighteone	45
		2,3-Dehydrokievitone	45
		Parvisoflavones-A	45
		1″,2″-Dehydrocyclokievitone	45
		7,2′-Dihydroxy-4′-methoxy-8-(3-methyl-2-butenyl) isoflavanone	45
	3-Hydroxyisoflavanones	Glycybridin J	45
	Isoflavans	Glabridin	81,83-86
		3′-Hydroxy-4′-*O*-methylglabridin	45
		8-Prenyl-phaseollinisoflavan	45
		Glycybridin H	45
	Hydroxyisoflavans	Licoricidin	81
	Isoflavone glycosides	Ononin	54
	Isoflavenes	Glycybridin D	45
		Glycybridin E	45
	Isoflavene derivatives	3,4-Didehydroglabridin	45
	Hydroxyisoflavonoids	Isoglycycoumarin	82
	Chalcones and chalcone derivatives	Isoliquiritigenin	57,81,82
		Isoliquiritin	54,57,82
		Licochalcone A	45,81,82
		Licuroside (neoisoliquiritin apioside)	87,88
		Isoliquiritin	81,87
		2-(5-*p*-Coumaryl apiosyl), isoliquiritin	87
		Licuraside (isoliquiritin apioside)	54,81,88

		Kanzonol Y	45
		[6″,6″-Dimethylpyrano(2″,3″:4,5)]-3′-γ,γ-dimethylallyl-2′,3,4′-trihydroxychalcone	45
		Licoagrochalcone A	45
		Isobavachalcone	45
		Kanzonol B	45
		Paratocarpin B	45
	α-Hydroxydihydrochalcones	Glycybridin A	45
		Glycybridin B	45
		Glycybridin C	45
	Chalcone oligoglycosides	Isoliquiritin apioside	81,87
	Flavonoids	Apigenin-6,8-di-C-glucoside (vicenin-2)	88
		Apigenin 2″-*O*-pentosyl-6-C-hexoside	88
		Methylapigenin *O*-hexoside	88
		Liquiritigenin apiosyl-glucoside isomers	88
		Naringenin-7-*O*-apiosyl-glucoside	88
		Euchrenone a_5	45
		Abyssinone II	45
		Kanzonol C	45
	Flvonols	(2*R*,3*R*)-3,4′,7-Trihydroxy-3′-prenylflavane	45
	Flavones	Apigenin	80
	Flavone glucosides	Vitexin	89
	Flavanones	Liquiritin	54,57, 81,82
		Liquiritigenin	54,57,81,82,87
		Naringenin	45
		Xambioona	45
		Shinflavanone	45
		3-Hydroxyglabrol	45
	Monohydroxyflavanones	(2*S*)-Abyssinone 1	45
	3-Hydroxypyranoflavanones	Kanzonol Z	45
	2-Arylbenzofuran flavonoids	Glyinflanin H (glabrocoumarone B)	45
		Kanzonol U	45
	2-Arylbenzofurans	Glycybridin F	45
		Glycybridin G	45
	Flavonoid glycosides	Liquiritin apioside	54,81,87
		Neoliquiritin apioside	87
	6α,11α-Pterocarpenes	Dehydroglyceollin I	45
	Pterocarpans	Licoagrocarpin	45
		Phaseollin	45
	3-Phenoxychromone	Glycybridin I	45
	3-Arylcoumarin	Glycycoumarin	81,82
		Glycybridin K	45
	Pyrano-3-arylcoumarin	Kanzonol W	45
	Coumestan derivatives	Isoglycyrol	82
	Phenolics	2′-*O*-Demethybidwillol	45
		Erybacin	45
	Miscellaneous compounds	*p*-Hydroxybenzylmalonic acid	81
		Glycyrrhizic acid monoammonium salt	54
Root culture	Triterpenes	Betulinic acid	90
		β-Amyrin	90

		Lupeol	90
Hairy root culture	Sesquiterpene lactones	Odoratin	91
	Isoflavones	Afrormosin	91
	Isoflavans	4'-*O*-Methylglabridin	92
	Dipyranoisoflavans	Glyinflanin K	92
	Chalcones and chalcone derivatives	Echinatin	91
		Kanzonol B	93
		4-Hydroxylonchocarpin	93
	Flavanones	Glabrol	93
		Xambioona	93
		3-Hydroxyglabrol	93
	Flavonoids	Isobavachalcone	93
		Euchrenone a5	93
		Abssinone II	93
		Kanzonol D	91
	Prenylated biaurone	Licoagrone	91
	Prenylated flavonoids	Licoagrochalcone A	93
		Licoagrocarpin	93
		Glabridin	92
		Tenuifolin B	92
		Lespedezaflavanone B	92
	Coumestans	Phaseol	91
	Others	Licoagrodione	92
Whole plant	Isoflavone	Genistein	94
	Chalcones	Echinatin	94
	Flavanones	Liquirtigenin	95
	Flavonoids	6-Aldehydo-isoophiopogonone	95
	3-Arylcoumarins	Licopyranocoumarin	94
	Polyphenols	Ellagic acid	95
	Phenolic compounds	Glicoricone	94
		Licofuranone	94

Table S2. Secondary metabolites in *Paeonia lactiflora.*

Part	Class	PSM	References
Root	Monoterpene glycosides	6-*O*-β-D-Glucopyranosyl-lactinolide	96
		Oxybenzoyl-paeoniflorin	96,97
		Albiflorin R1	98
		Paeoniflorin	97,99-110
		Albiflorin (pinnae-type monoterpenes)	97,99-102,104-110
		Benzoylpaeoniflorin	97,99,101,104,108-110
		Oxypaeoniflorin/oxypaeoniflorin isomer	97,110
		Lactiflorin	97,108,110
		3-*O*-Methylpaeoniflorin	97
		Mudanpioside J	97
		Paeoniflorin sulfonate	99
		6'-*O*-β-D-Glucopyranosylalbiflorin	100
		6'-*O*-Benzoylalbiflorin	100
		Benzoyl paeoniflorin	100,107
		Isopaeoniflorin	111
		Isobenzoylpaeoniflorin	111
		4-*O*-Methyl-paeoniflorin	108,111
		1-*O*-β-D-Glucopyranosyl-8-*O*-benzoylpaeonisuffrone	112
		Paeonidanin	104,112
		Paeonilactone-B	101
		Paeonilactone-C	101
		Paeoniflorigenone	101,105

		Oxypaeoniflorin	101,108
		4'-*O*-Benzoylpaeoniflorin	113,114
		4-*O*-Galloylalbiflorin	103,105,113,114
		Paeonin A	115
		Paeonin B	115
		Paeonin C	115
		8-Debenzoylpaeonidanin	115
		Galloylalbiflorin	103,104
		Lalbiflorin	103
		Galloy paeoniflorin	103
		6-*O*-Galloyl-β-D-glucopyraneose	103
		4-Methylpaeoniflorin	104
		Paeonidanin A	104
		Benzoylalbiflorin	104
		Debenzoylalbiflorin	104
		2'-*O*-Benzoylpaeoniflorin	116
		Albiflorin R2	116
		Albiflorin R3	116
		Nor-paeonilactone	105
		Paeonilactone D	105
		Paeonilactone A	105
		9-Hydroxypaeonilactone A	105
		Paeonin D	105
		6′-*O*-Galloylalbiflorin	105
		Galloylpaeoniflorin	105,108
		Paeonidanin F	117
		Paeonidanin G	117
		Paeonidanin H	117
		Desbenzoylpaeoniflorin	110
		Pinen-vicianoside	110
		Hydroypaeoniflorin	107
		8-Debenzoylpaeoniflorin	118
		Paeonidanin B	118
		Paeonidanin C	118
		Paeonidanin D	118
		Paeonidanin E	108
		Oxypaeoniflora	109
	Monoterpenes	1-*O*-β-D-Glucopyranosylpaeonisuffrone	96,112
		Lactinolide	96
		Paeonilactinone	96
		4-Methylbenzoylpaeoniflorin	104
		Paeoniphenoside	104
		Paeonisuffrone C	105
		Paeonisuffrone	105
		6′-*O*-Acetylpaeoniflorin	106
		Galloyl-desbenzoylpaeoniflorin	110
		Galloylpaeoniflorin/galloylalbiflroin	110
		Isomaltopaeoniflorin/glucopyranosylalbiflorin	110
		Glucopyranosyl-enzoylpaeonisuffrone	110
		Di-*O*-galloylpaeoniflorin	110
		Pyrethrin I	118
		Pyrethrin II	118
		Paeoniflorol	108
		4'-Hydroxypaeoniflorigenone	108
		4-*epi*-Albiflorin	108
		Paeonivayin	108
		Salicylpaeoniflorin	108
		Mudanpioside C	108

		Mudanpioside J	108
		Benzoyloxypaeoniflorin	108
		6'-*O*-Vanillyloxypaeoniflorin	108
		Paeonenoide D	119
		Paeonenoide E	119
	Triterpenes	β-Sitosterol	103,120
		Palbinone	105
		Oleanic acid	107,119
		11α,12α-Epoxy-3β,4β-dihydroxy-24-norolean-28-oic acid	119
		3β,4β,23,29-Tetrahydroxy-24-norolean-12-en-28-oic acid	119
		Paeonenoide A (24,30-dinortriterpenoid)	119
		11α,12α-Epoxy-3β,23-dihydroxyolean-28,13β-olide	119
		11α,12α-Epoxy-3β-hydroxyolean-28,13β-olide	119
		Hederagenin	119
	Phytosterols	Daucosterol (β-sitosterol glucoside)	118
	Phenolic acids	(+)-Catechin-7-*O*-gallate	108
		(-)-Epicatechin-7-*O*-gallate	108
	Phenolic glycosides	2-Methoxy-5-(*E*)-propenyl-phenol-β-vicianoside	122
	Phenolic aldehydes	Paeonalin A	120
	Phenolic compounds	Phloroglucinol	123
		Resorcinol	123
		Catechol	123
		Methyl gallate	103,106,108
		Pyrogallol	103
		1,2,3-Benzenetriol	104
		Bisphenol A (diphenylmethane derivatives and bisphenols)	106
		Phenol (carbolic acid)	118
	Polyhenols	1,2,3,4,6-Pentagalloyl glucose (PGG)	99,103,108
		Paeonol	102,106-108,124
		3-*O*-Galloylquinic acid	118
		4-*O*-Galloylquinic acid	118
		Pedunculagin	118
		1,2,3,6-Tetra-*O*-galloyl-β-D-glucose	118
		1,3,6-Trigalloyl-β-D-glucose	118
		1,2,3-Tri-*O*-galloyl-β-D-glucose	118
		1,2,6-Tri-*O*-galloyl-β-D-glucose	118
	Hydrolysable tannins	Casuariin	118
		Casuarictin	118
		5-Desgalloylstachyurin	118
		1-*O*-Galloyl-β-D-glucose	118
		Strictinin	118
		Tellimagrandin I	118
		2,3-*O*-(*S*)-Hexahydroxydiphenoyl-D-glucopyranose	118
	Gallotannins	Pentagalloylglucose	110
		Hexagalloylglucose	110
		Heptagalloylglucose	110
		Octagalloylglucose	110
		Nonagalloylglucose	110
	Flavonoids	(+)-Catechin	102-104,107-109
		4',5-Dihydroxyflavanone-7-*O*-β-D-glucoside	104
		5,7-Dihydroxyflavanone-4'-*O*-β-D-glucoside	104
		Narigenin chalcone 2'-*O*-xyloside	108
		4,2',4',6',7,8-Hexahydroxy-7,(8)-dihydro-chalcone, (cilicicone-A)	108
	Prenylated furocoumarins	Pangelin	125
	Carboxylic	Gallic acid	102-104,107-109,124

	acids	Benzoic acid	102,106,108,109,120
		Vanillic acid	104
		3,5-Dihydroxy-4-methoxyl-benzoic acid	108
	Esters	Ethyl gallate	103
		Di-(2-ethylhexyl)phthalate	103
		1,2,3,4,6-Penta-*O*-galloyl-β-D-glucopyranose	106,124
		Methyl 4-hydroxy-3-methoxybenzoate	108
	Anthocyanins	Peonin (peonidin-3,5-diglucoside)	118
	Miscellaneous compounds	Paeonalin B (volatile crystalline substance)	120
		Benzoylsucrose	110
		Galloylsucrose	110
Flower	Monoterpene glycosides	Paeoniflorin	126,127
		Albiflorin	102,126,127
		Oxypaeoniflorin	127
	Flavonoids	Pelargonidin 3-glucoside	123
		Cyanidin 3,5-diglucoside	123
		Peonidin 3-glucoside	123
		Peonidin 3,5-di-*O*-β-D-glucopyranoside	123
		Malvoside (petal)	123
		Catechin	102
		Quercetin-3-*O*-glucoside-6″-gallate	126
		Kaempferol-3-*O*-glucoside-6″-gallate	126
		Kaempferol-3,7-di-*O*-β-D-glucoside	126
		Quercetin-3-*O*-(6''-*O*-galloyl)-glucoside	128
		Quercetin-3-*O*-β-D-glucoside	128
		Kaempferol-3-*O*-(6''-*O*-galloyl)-glucoside	128
		Isohamnetin-3-*O*-β-D-glucoside	128
		Kaempferol	128
		Kaempferol-3-*O*-β-D-glucoside	128
		Kaempferol-7-*O*-β-D-glucoside	128
		Quercetin-3-*O*-(6″-*O*-galloyl)-glucoside	127
		Kaempferol-3,7-di-*O*-β-D-glucopyranoside	127
		Kaempferol-3-*O*-β-D-glucopyranosyl-7-*O*-*α*-L-rhamnopyranoside	127
		Kaempferol-3-*O*-*β*-D-galactopyranosyl-7-*O*-β-D-glucopyranoside	127
		Kaempferol 3-*O*-(6-*O*-galloyl)-β-D-glucopyranoside	127
		Astragalin	127
		2-Phenylethyl-[*α*-L-rhamnopyranosyl-(1→6)]-β-D-glucopyranoside	127
	Tannins	1,2,3,6-*tetra*-*O*-Galloyl-β-D-glucopyranoside	126,127
		1,2,3,4,6-*penta*-*O*-Galloyl-β-D-glucopyranoside	126,127
	Hydrolysable tannins	1-*O*-Galloyl-β-D-glucose	126
	Gallotannins	1,2,3,4,6-Pentagalloyl-β-D-glucose	128
	Phenolic compounds	Phenethyl alcohol	123
		Methyl gallate	126,127
		Ethyl gallate	126,127
		An equilibrium mixture of methyl *m*-digallate and methyl *p*-digallate	127
		An equilibrium mixture of ethyl *m*-digallate and ethyl *p*-digallate	127
	Carboxylic acids	Gallic acid	126,127
		Vanillic acid	126,127
	Esters	1-*O*-Galloyl-β-D-glucopyranoside	127
	Miscellaneous compounds	(Z)-Hex-3-en-1-ol	123
		13-Methyltetradecanoic acid	123
		(Z)-Hex-3-enal	123
		1′-*O*-Galloylsucrose	127
		6′-*O*-Galloylsucrose	127
		6-*O*-Galloyl-D-glucopyranoside	127
		3-Hydroxycitronellic acid 3-*O*-*β*-D-glucopyranoside	127
		2-Phenylethyl)]-β-D-glucopyranosyl-(1→6)]-β-D-glucopyranoside	127

		2-Phenylethyl-β-D-glucopyranoside	127
		3-*O*-β-D-Glucopyranoside	127
		6-*O*-*m*-Digalloyl-1,2,3,4-*tetra*-*O*-galloyl-β-D-glucopyranoside	127
Leaf	Monoterpene glycosides	Albiflorin	102
		Paeoniflorin	102
	Flavonoids	Catechin	102
	Carboxylic acids	Gallic acid	102
		Benzoic acid	102
	Phenolic compounds	Paeonol	102
Stem	Monoterpene glycosides	Albiflorin	102
		Paeoniflorin	102
	Flavonoids	Catechin	102
	Carboxylic acids	Gallic acid	102
		Benzoic acid	102
	Phenolic compounds	Paeonol	102
Fruit	Monoterpene glycosides	Albiflorin	102
	Flavonoids	Catechin	102
	Dimeric ellagitannins	Paeonianin A	129
		Paeonianin B	129
		Paeonianin C	129
		Paeonianin D	129
	Ellagitannin monomer	Paeonianin E	129
	Carboxylic acids	Gallic acid	102
		Benzoic acid	102
	Phenolic compounds	Paeonol	102
Rhizome	Monoterpene glycosides	Albiflorin	102
		Paeoniflorin	102
	Flavonoids	Catechin	102
	Carboxylic acids	Gallic acid	102
		Benzoic acid	102
	Phenolic compounds	Paeonol	102
Root and rhizome	Triterpenes	Oleanolic acid	130
		Ursolic acid	130
Seed	Monoterpene glycosides	Paeoniflorin	102,131
		Albiflorin	102,131
		Oxypaeoniflorin	131
		4″-Hydroxyl-albiflorin	131
		Paeonidanin	131
		Albiflorin R1	131
		4-*O*-Methyl-paeoniflorin	131
		Oxypaeonidanin	131
	Flavonoids	Luteolin	132
		Catechin	102
	Carboxylic acids	Benzoic acid	102
	Stilbenes	*cis*-ε-Viniferin	133
		trans-ε-Viniferin	132-134
		Resveratrol	132,133
		Gnetin H	132,133
		Suffruticosol A	132-134
		Suffruticosol B	133,134
		trans-Resveratrol-4'-*O*-beta-D-glucopyranoside	133
		trans-Gnetin H	134

Table S3. Secondary metabolites in *Paeoia veitchii.*

Part	Class	PSM	References
Root	Monoterpene glycosides	Acetoxypaeoniflorin	135
		Paeoniflorin	136-138
		Albiflorin (pinnae-type monoterpenes)	136,137
		Benzoylpaeoniflorin	136,137
		Hydroxypaeoniflorin	137
		Benzoylhydroxypaeoniflorin	137
		8-Debenzoylpaeoniflorin	118
		Lactiflorin	118
		Oxypaeoniflorin	118
		Paeoniflorigenone	118
		(*Z*)-(1*S*,5*R*)-β-Pinen-10-yl-β-vicianoside	118
		4-*O*-Methyloxypaeoniflorin	138
		4-*O*-Ethylpaeoniflorin	138
		Paeonidanin	138
		9-Ethyl-neo-paeoniaflorin A	138
		4-*O*-Methyl-paeoniflorin	108,138
		Paeonidanin I	139
		Paeonidanin J	139
		Paeonidanin K (dimeric monoterpene glycoside)	139
		Galloylpaeoniflorin	140
		Oxypaeoniflorin	140
	Monoterpenes	Paeonisothujone	137
		Paeoveitol A	141
		Paeoveitol B	141
		Paeoveitol C	141
	Triterpenes	Akebonic acid	142
		(3β,4β,11α,12α,13β)-11,12-Epoxy-4,13-dihydroxy-3,23-(isopropylidenedioxy)-24,30-dinorolean-20(29)-en-28-oic acid 28,13-lactone	142
		(3β,4β,11α,12α,13β)-11,12-Epoxy-3,13-dihydroxy-4,23-(isopropylidenedioxy)-24,30-dinorolean-20(29)-en-28-oic acid 28,13-lactone	142
		(3β,4α,11α,12α,13β)-11,12-Epoxy-13-hydroxy-3,23-isopropylidenedioxy-30-norolean-20(29)-en-28-oic acid 28,13-lactone	142
		(3β,4β)-4-Hydroxy-3,23-(isopropylidenedioxy)-24,30-dinorolean-12,20(29)-dien-28-oic acid	142
		Mudanpinoic acid A	137
		β-Sitosterol	118
	24,30-Dinortriterpenoids	Paeonenoide A	142
		Paeonenoide B	142
		Paeonenoide C	142
	Norditerpenes	(±)-Paeoveitol	143
	Phytosterol	Daucosterol (β-sitosterol glucoside)	118
	Flavonoids	Catechin	136,140
	Hydrolysable tannins	Tellimagrandin II (eugeniin)	118
	Carboxylic acids	Gallic acid	136,144
		Benzoic acid	136,140
	Phenolic glycoside	2-*O*-[α-L-Arabinopyranosyl-(1→6)-β-D-glucopyranoside]-benzaldehyde	144
	Phenolic compounds	Pentagalloylglucose	136,144
		Isosalicin	144
		Salicin	144
		Salicyl alcohol	144
		Methyl gallate	144
		Paeonol	144
		2-Hydroxybenzyl alcohol	137
	Polyphenols	1,2,3,6-tetra-*O*-galloyl-β-D-glucose	118
	Esters	β-Sitosterol linoleate	140

		Daucosterol linoleate	140
	Benzofurans	Paeoveitol D	141
		Paeoveitol E	141
	Miscellaneous compounds	Arbutin	140
		His(2-hydroxybenzyl) ether	137
Flower petal	Anthocyanin	Cyanidin-3-*O*-glucoside-5-*O*-galactoside	145
		Pelargonidin-3-*O*-glucoside-5-*O*-galactoside	145

Table S4. Secondary metabolites in *Eriobotrya japonica.*

**Part**	**Class**	**PSM**	**References**
Leaf	Triterpenes	Ursolic acid	146-155
		Euscaphic acid	146,147,149,150,152,155-157
		Maslinic acid	146,149,150,152,153,157,158
		Oleanolic acid	149,150, 152,153,155-157
		Corosolic acid	150,152-154,157-159
		3-*O*-*cis*-*p*-Coumaroyltormentic acid	150,154,157,160
		3-*O*-*trans*-*p*-Coumaroyltormentic acid	150,154,157,160
		2α-Hydroxyursolic acid	147-149
		Tormentic acid	152-154,158
		Methyl maslinate	146
		2α,3α-Diacetoxy-19α-hydroxyurs-12-en-28-oic acid	146
		Maslinic acid diacetate	146
		Methyl betulinate	150,152,157
		β-Sitosterol	154,161
		2α,3α,19α-Trihydroxyurs-12-en-28-oic acid	162,163
		Methyl ursolate	150,157
		δ-Oleanolic acid	150,157
		Betulinic acid	150,157
		2α,3α,19α-Trihydroxyolean-12-en-28-oic acid	153,158
		2α,3α-Dihydroxyursolic acid	153,158
		3β,6α,19α-Trihydroxyurs-12-en-28-oic acid	147
		3,6,19-Trihydroxyurs-12-en-28-oic acid	164
		2,3-Dihydroxyurs-12-en-28-oic acid	164
		23-*trans*-*p*-CoumaroyItormentic acid	162
		23-*cis*-*p*-Coumaroyltormentic acid	162
		3-*O*-*trans*-Caffeoyltormentic acid	162
		3-*O*-*trans*-*p*-Coumarolyrotundic acid	162
		3β,6β,19α-Trihydroxyurs-12-en-28-oic acid	162
		2α,3β,19α,23-Tetrahydroxyurs-12-en-28-oic acid	162
		3-*O*-*trans*-Feruloyl euscaphic acid	165
		Pomolic acid	149
		2α,3α,19α-Trihydroxy-12-oleanen-28-oic acid	156
		2α-Hydroxyoleanolic acid	156
		Methyl arjunolate	150
		2α, 3α,23-Trihydroxyolean-12-en-28-oic acid	150
		3-Epicorosolic acid	150
		1β-Hydroxyeuscaphic acid	150
		Usrolic acid lactone	150
		2β, 3β, 19α-Trihydroxyurs-12-en-28-oic acid	163
		Methyl corosolate, (methyl 2α-3α-dihydroxyurs-12-en-28-oate)	152
		Arjunolic acid	158
		Methyl 2α-hydroxyursolate	158
		Urs-12-en-28-oic acid,3-hydroxyl-methyl ester (3α)	166
		Squalene	166
		α-Hydroxyoleanolic acid	155
		Arjunic acid	155
		3α-*trans*-Feruloyloxy-2α-hydroxyurs-12-en-28-oic acid	161

		3-*O*-*trans*-Feruloyleuscaphic acid	161
	Sesquiterpene glycosides	Loquatifolin A	167
		Nerolidol-3-*O*-α-L-rhamnopyranosyl-(1→2)-β-D-glucopyranoside	168
		Nerolidol-3-*O*-α-L-rhamnopyranosyl-(1→4)-α-L-rhamnopyranosyl-(1→2)-β-D-glucopyranoside	168
		Nerolidol-3-*O*-α-L-rhamnopyranosyl-(1→4)-α-L-rhamnopyranosyl-(1→6)-β-D-glucopyranoside	168
		Isohumbertiol-3-*O*-{α-L-rhamnopyranosyl-(1→4)-α-L-rhamnopyranosyl-(1→2)-{α-L-rhamnopyranosyl-(1→6)]}-β-D-glucopyranoside	169
		Isohumbertiol-3-*O*-{α-L-rhamnopyranosyl-(1→4)-α-L-rhamnopyranosyl-(1→2)-{α-L-(4-trans-feruloyl)-rhamnopyranosyl-(1→6)]}-β-D-glucopyranoside	169
		Nerolidol-3-*O*-α-L-rhamnopyranosyl-(1→4)-α-L-rhamnopyranosyl-(1→2)-[α-L-(4-trans-feruloyl)-rhamnopyranosyl-(1→6)]-β-D-glucopyranoside	169,170
		Nerolidol-3-*O*-α-L-rhamnopyranosyl-(1→6)-β-D-glucopyranoside	171
		Nerolidol-3-*O*-α-L-rhamnopyranosyl-(1→2)-[α-L-rhamnopyranosyl-(1→6)]-β-D-glucopyranoside	171
		β-Monocyclonerolidol-3-*O*-α-L-rhamnopyranosyl-(1→4)-α-L-rhamnopyranosyl-(1→2)-[α-L-rhamnopyranosyl-(1→6)]-β-D-glucopyranoside	172
		Nerolidol-3-*O*-α-L-rhamnopyranosyl-(1→4)-α-L-rhamnopyranosyl-(1→2)-[α-L-rhamnopyranosyl-(1→6)]-β-D-glucopyranoside	163,168,170,173
	Sesquiterpenes (essential oil)	α-Ylangene	174
		α-Farnesene	174
		β-Farnesene	174
		α-Cadinol	174
		α-Bisabolol	175
	Monoterpenenes (fenchane monoterpenoids)	Vomifoliol	158
	Monoterpenenes (essential oils)	β-Pinene	174
		Camphene	174
		β-Mycrene	174
		ρ-Cymene	174
		Linalool	174
		trans-Linalool oxide	174
		Camphor	174
		Nerol	174
		α-Pinene	175
		Limonene	175
	Flavonoids	Quercetin-3-rhamnoside	148
		Quercetin-3-sambubioside	148,160
		Kaempferol 3-*O*-rhamnoside	148,176
		Quercitrin	177
		Afzelin	177
		Procyandin B-2	178,179
		Procyanidin C-1	178,179
		Procyanidin oligomer	178,179
		(2S)- and (2R)-Naringenin	178
		8-*C*-α-L-Rhamnopyranosyl-(1→2)-β-D-glucopyranosides	178
		Cinchonain Id 7-*O*-β-D-glucopyranoside	178
		Cinchonain Ia	178
		(-)-Epicatechin	178
		cinchonain Ib	178

		Cinchonain Ic	178
		Cinchonain Id	178
		Epicatechin-(4β→2)-phloroglucinol	178
		(-)-Epigallocatechin gallate	178
		Quercetin 3-*O*-sophoroside	178
		Cinchonain Id 7-*O*-glucopyranoside	179
		Cinchonain IIb	179
		(2*S*)-Naringenin 8-*C*-rha (1''→2')-glucopyranoside	179
		Kaempferol 3-*O*-α-L-(2'',4''-di-*E*-feruloyl)-rhamnoside	180
		Kaempferol 3-*O*-α-L-(2'',4''-di-*Z*-*p*-coumaroyl)-rhamnoside	180
		Kaempferol 3-*O*-α-L-(2'',4''-di-*E*-*p*-coumaroyl)-rhamnoside	180,181
		Quercetin 3-*O*-glucoside	176
		Quercetin 3-*O*-galactoside	176
		Kaempferol 3-*O*-glucoside	176
		Kaempferol 3-*O*-galactoside	176
		A kaempferol 3-*O*-dihexoside (probably the 3-*O*-sophoroside)	176
		Kaempferol 3-*O*-neohesperidoside	176
		Kaempferol 3-*O*-rutinoside	176
		Isorhamnetin 3-*O*-glucoside	176
		Isorhamnetin 3-*O*-galactoside	176
		Quercetin 3-*O*-rutinoside (rutin)	163,176
		Quercetin-3-*O*-β-D-glucoside	156
		Quercetin-7-α-L-rhamnoside	156
		Rhamnocitrin	158
		Quercetin-4′-*O*-β-D-galactoside	158
		Kaempferol-3-*O*-α-L-(3''-*Z*,4''-*E*-di-*p*-coumaroyl)-rhamnoside	181
		Kaempferol-3-*O*-α-L-(3'',4''-di-*E*-*p*-coumaroyl)-rhamnoside	181
		Kaempferol-3-*O*-α-L-(2''-*E*-feruloyl,4''-*E*-pcoumaroyl)-rhamnoside	181
		Kaempferol-3-*O*-α-L-(2''-*E*-p-coumaroyl,4''-*E*-feruloyl)-rhamnoside	181
		Naringenin-8-C-rhamnoglucoside	160
		Kaempferol 3-*O*-sophoroside	160
		Kaempferol 3-*O*-rhamnosyl glucoside-7-*O*-rhaminoside	160
		Quercetin 3-*O*-glucosylrhamnosyl-glucoside	160
		Cinchonain glucoside + (sodium)	160
	Ionone-derived glycosides	Vomifoliol-9-*O*-β-D-glucopyranoside (roseoside)	169,182
		3-oxo-α-Ionyl-9-*O*-β-D-glucopyranoside	169
		3-oxo-a-Ionyl-9-*O*-β-D-apiofuranosyl-(1→6)-β-D-glucopyranoside	169
		Vomifoliol-9-*O*-β-D-apiofuranosyl-(1→6)-β-D-glucopyranoside	169
		(6*S*,9*R*)-Roseoside	179
	Glycosides	Schaftoside (*C*-glycoside)	176
		Arbutin	182
		Amygdalin	183
	Phenylpropanoids	Ferulic acid	170
		Phenylpropyl acid	163
		Chlorogenic acid	148
		Methyl chlorogenate	148
		Eugenyl β-rutinoside	158
	Megastigmane glycosides	Eriojaposide B	161
		(6*R*,9*R*)-3-oxo-α-Ionyl-9-*O*-α-apiofuranosyl-(1''→6')-*O*-β-glucopyranoside	161
		Citroside A	161
		Eriojaposide A	160,161,179
		(6*S*,9*R*)-Vomifoliol-9-*O*-β-apiofuranosyl-(1''→6')-*O*-β-glucopyranoside	161,179
		(6*S*,9*R*)-Vomifoliol-9-*O*-β-xylopyranosyl-(1''→6')-*O*-β-	161,179

		glucopyranoside	
		(6*R*,9*R*)-3-oxo-α-Ionyl-9-*O*-β-glucopyranoside	161,179
		(6*R*,7*E*,9*R*)-9-Hydroxy-4,7-megastigmadien-3-one 9-*O*-β-D-apiofuranosyl-(1→6)-β-D-glucopyranoside	156
		(6*R*,7*E*,9*R*)-9-Hydroxy-4,7-megastigmadien-3-one 9-*O*-β-D-xylopyranosyl-(1→6)-β-D-glucopyranoside	156
		(6*R*,7*E*,9*R*)-9-Hydroxy-4,7-megastigmadien-3-one *9-O*-α-L-arabinopyranosy-(1→6)-β-D-glucopyranoside	156
		(6R,7E,9R)-9-Hydroxy-4,7-megastigmadien-3-one	156
		(6*R*,7*E*,9*R*)-9-Hydroxy-4,7-megastigmadien-3-one 9-*O*-β-D-glucopyranoside	156
		(6*R*,7*E*,9*S*)-9-Hydroxy-4,7-megastigmadien-3-one 9-*O*-β-D-gluco-pyranoside	156
		(6*S*,7*E*,9*R*)-6,9-Dihydroxy-4,7-megastigmadien-3-one	156
		(6*S*,7*E*,9*R*)-6,9-Dihydroxy-4,7-megastigmadien-3-one 9-*O*-β-D-glucopyranoside	156
	Lignans	Linguersinol	156
		2,6-Dimethoxy-4-(2-propenyl)phenol	156
		2,6-Dimethoxy-4-(2-propenyl)phenol 1-*O*-β-D-glucopyranoside	156
	Essential oils	*cis*-β,γ-Hexenol	174
		Tartaric acid	174
		1-Pentene-3-ol	175
		(*E*)-3-Penten-2-one	175
		Toluene	175
		Furfural	175
		2-Methylbutene	175
		2-Hexenal	175
		Ethylbenzene	175
		1-Hexanol	175
		1,3-Dimethylbenzene	175
		3-Methyl-1-butyl acetate	175
		2,6-Lutidine	175
		1,4-Dimethylbenzene	175
		α, β-Angelica lactone	175
		Benzaldehyde	175
		6-Methyl-hept-5-en-2-one(methyl heptenone)	175
		2,4-Heptadienal	175
		4-Methyl-1-cyclohexene	175
		2-Hexenoic acid	175
		Benzyl alcohol	175
		2-Phenylacetaldehyde	175
		γ-Hexalactone	175
		3,7-Dimethyl-1,6-octadien-3-ol	175
		Nonanal	175
		Phenylethyl alcohol	175
		2,6,6-Trimethyl-*2*-cyclohexene-*1,4*-dione	175
		Hexanoic acid, 2-methyl-, ethyl ester	175
		Safranal	175
		Dihydrocoumarins	175
		(+)-Carvone	175
		2-Hexanoylfuran	175
		Geraniol	175
		4-Methoxybenzaldehyde	175
		Cinnamaldehyde	175
		Butenyl cyclohexene	175
		1-Methoxy-4-(2-propenyl)-benzene	175
		Indole	175
		2-Methoxy-4-vinylphenol	175

		Geranic acid (pheromones)	175
		β-Damascone	175
		α-Ionone	175
		1,4-Dimethoxy-2,3-dimethylbenzene	175
		Geranylacetone	175
		β-Ionone	175
		β-Bisabolene	175
		2,6-Di-tert-butyl-4-methylphenol	175
		Dihydroactinidiolide	175
		Elemicin	175
		(*E*)-Nerolidol	175
		Caryophyllene	175
		Butyl benzoate	175
		2,6-Dimethoxy-4-(2-propenyl)-phenol	175
		Farnesol	175
		Farnesyl acetate	175
		Methyl benzoate	175
		2,3-Biphenyl-2-cyclopropene-1-one	175
		6,10,14-Trimethyl-2-pentadecanone	175
		Diisobutyl phthalate	175
		Palmitic acid ethyl ester	175
		9,12,15-Octadecatrien-1-ol,(Z,Z,Z)-	175
	Essential oils (aromatic compounds)	Thymol	175
		Myristicin	175
	Esters	Benzoic acid methyl ester	166
		Linoleic acid, ethyl ester	160
	Carboxylic acids	Gallic acid	178
	Phenols	Ellagic acid	178
	Miscellaneus compounds	A-type dimeric procyanidin	160
		9-*O*-Apiosyl (1–6) glucoside	166
		Neophytadiene	166
		Dibenzofuran (phytoalexins)	176
Flower	Triterpenes	Ursolic acid	184-186
		Oleanolic acid	184-186
		2α,3α,19α-Trihydroxyurs-5,12-dien-28-acid	184
		2β,3β,23α-Trihydroxyolean-12-en-28-acid	184
		β-Sitosterol	186
		3β,19α-Trihydroxyursolic-4-aldehyde-12-en-28-acid	186
		β-Daucosteriol	186
		Maslinic acid	186
		2α-Hydroxyursolic acid	186
	Sesquiterpenes	Nerolido	187
	Flavonoids	Hesperetin	188
		Quercetin-3-*O*-α-L-galactoside	186
	Glycosides	Amygdalin	183,185,189
	Phenols	Gallic acid	188
	Aldehydes	Hexadecanal	187
		Octadecanal	187
		p-Anisaldehyde	187,190
	Ketones	6, 10, 14-trimethyl-2-pentadecanone	187
	Esters	4-Methoxy methylbenzoate	187
		4-Methoxy ethylbenzoate	187
		Phenyl ethyl octanoate	187
		Methyl hexadecanoate	187
		Methyl hexadecanoate	187
		Methyl linoleate	187
		3-Cyclopentylpropionic acid, decyl ester	190

		Hexadecanoic acid, ethyl ester	190
		Phenylalanin, 4-amino-N-t-butyloxycarbonyl-, t-butyl ester	190
		Benzoic acid, 4-methoxy-, methyl ester	190
		Benzoic acid, 4-methoxy-, ethyl ester	190
		4-Methoxybenzoic acid, allyl ester	190
		Tridecanoic acid, 12-methyl-, methyl ester	190
		Dibutyl phthalate	190
		Geranyl isovalerate	190
		Cyclopropanebutanoic acid, 2-[[2-[[2-[(2-pentylcyclopropyl) methyl]cyclopropyl] methyl] cyclopropyl] methyl]-, methyl ester	190
		Butyl octyl phthalate	190
		Benzyl butyl phthalate	190
		Hexadecanoic acid, ethyl ester	190
		Retinoic acid, methyl ester	190
		Linoleic acid ethyl ester	190
		1,2-Benzenedicarboxylic acid, butyl octyl ester	190
	Organic acids	Quinic acid	191
		Tartaric acid	191
		Citric acid	191
		Oxalic acid	191
		cis-Aconitic acid	191
		Malic acid	191
	Miscellaneus compounds	Butylated hydroxytoluene	187
		5,7,9(11)-Androstatriene,3-hydroxy-17-oxo-	190
		Benzoylformic acid	190
		NOPOL	190
		1,3-Dioxane,5-(hexadecyloxy)-2-pentadecyl-	190
		1-Hexadecanol, 2-methyl-	190
		5α-Cholestan-2-one,oxime	190
		Ethanone,1-(5,6,7,8-tetrahydro-2,8,8-trimethyl-4H-cyclohepta[b]furan-5-yl)-	190
		Tert-hexadecanethiol	190
		1-Chloroeicosane	190
		1-Eicosene	190
		Ethanol, 2-(octadecyloxy)-	190
		Androst-5,7-dien-3-ol-17-one	190
		Propanoic acid, 2-(3-acetoxy-4,4,14-trimethylandrost-8-en-17-yl)-	190
		9-Octadecene, 1-[2-(octadecyloxy)ethoxy]-	190
		Heptadecane, 9-hexyl-	190
		Benzoyl bromide	190
		Methyl N-(N-benzyloxycarbonyl-beta-l-aspartyl)-β-d-glucosaminide	190
		Phthalaldehydic acid	190
		Heptadecane, 1-bromo-	190
		5-Hydroxymethyl-1,1,4α-trimethyl-6 methylenedecahydronaphthalen-2-ol	190
		2-Bromotetradecanoic acid	190
		E-8-Methyl-9-tetradecen-1-ol acetate	190
		Octadecane, 3-ethyl-5-(2-ethylbutyl)-	190
		7-Methyl-*Z*-tetradecen-1-ol acetate	190
		Benz[e]azulene-3,8-dione,3α,4,6α,7,9,10,10α,10β-octahydro-3α,10α-dihydroxy-5-(hydroxymethyl)-7-(1-hydroxy-1-methyl ethyl)-2,10-dimethyl-	190
		1-Hexadecanol, 2-methyl-	190
		psi,.psi.-Carotene, 1,1',2,2'-tetrahydro-1,1'-dimethoxy-	190
		(22*S*)-21-Acetoxy-6α,11α-dihydroxy-16α,17α-propylmethylenedioxypregna-1,4-diene-3,20-dione	190

		5,7,9(11)-Androstatriene, 3-hydroxy-17-oxo-	190
		Benzaldehyde	190
		3-Carene	190
		Pregn-4-en-18-oic acid, 11-(acetyloxy)-6,7-epoxy-9,20-dihydroxy-3-one-γ-lactone	190
		2-Hexadecanol	190
		Gibberellic acid	190
		Calarene epoxide	190
		3-Methoxymethoxy-2,2-dimethyloct-4-ene	190
		Octadecane, 3-ethyl-5-(2-ethylbutyl)-	190
		Pregn-4-ene-3,20-dione,17,21-dihydroxy-, bis(*O*-methyloxime)	190
		Bicyclo[4.3.0]nonane, 3-butyl-4-hexyl-	190
		l-(+)-Ascorbic acid 2,6-dihexadecanoate	190
		Ergosteryl acetate	190
		7αH-cyclopenta[α]cyclopropa[f]cycloundecene-2,4,7,7α,10,11-hexol, 1,1α,2,3,4,4α,5,6,7,10,11,11ɑ-dodecahydro-1,1,3,6,9-pentamethyl-,2,4,7,10,11-pentaacetate	190
	Carotenoids	Zeaxanthin	190
		Fucoxanthin	190
		Canthaxanthin	190
Stem bark	Triterpenes	β-Sitosterol	192
		β-Sitosterol-3-*O*-β-D-glucopyranoside	192
		Oleanolic acid	192
	Flavonoids	Catechin	192
		Cinchonain IIb	192
	lignans	Lyoniresinol	192
		Lyoniresinol 2α-*O*-β-D-xylopyranoside	192
Root	Glycosides	Amygdalin	183
Seed	Triterpenes	β-Sitosterol	193
	Flavonoids	Kaempferol	194
		Rutin	194
		Narigin	194
	Phenolic acids	3,4-Dihydroxybenzoic acid	194
		trans-Cinnamic acid	194
		5-Cafeoylquinic acid	194
		p-Coumaric acid	194
	Phenylpropanoids	Caffeic acid	195
		Chlorogenic acid	195
	Carboxylic acids	Benzoic acid	195
Fruit	Triterpenes	Ursolic acid	196
		Oleanolic acid	196
	Glycosides	Amygdalin	183
	Leucoanthocyanidin	Loquatoside	197
	Organic acids	Quinic acid	191
		Tartaric acid	191
		Citric acid	191,198
		Oxalic acid	191
		cis-Aconitic acid	191
		Malic acid	191,198
		Fumaric acid	198
		Succinic acid	198
	Phenolic compounds	Ortho-diphenol	198
		Chlorogenic acid	199
		Neochlorogenic acid	199
		Hydroxybenzoic acid	199
		5-*p*-Feruloylquinic acid	199
		Protocatechuic acid	199

		4-Caffeoylquinic acid	199
		Epicatechin	199
		O-Coumaric acid	199
		Ferulic acid	199
		p-Coumaric acid	199
	Carotenoids	β-Carotene	198,200
		Neo-pcarotene U	200
		Neo-β-carotene B	200
		Cryptoxanthin	198
	Alcohols	1-Propanol	201
		2-Methyl-1-propanol	201
		1-Butanol	201
		2-Methyl-2-butanol	201
		2-Pentanol	201
		3-Pentanol	201
		1-Penten-3-ol	201
		3-Penten-2-ol	201
		(*Z*)-2-Penten-1-ol	201
		1-Hexanol	201
		(*E*)-2-Hexen-1-ol	201
		(*E*)-3-Hexen-1-ol	201
		(*Z*)-3-Hexen-1-ol	201
		1-Heptanol	201
		1-Octanol	201
		2-Octanol	201
		1-Hexadecanol	201
		Citronelloll	201
		3,7-Dimethyl-1,5,7-octa-triene-3-ol (hotrienol)	201
		α-Terpineol	201
		Benzylalcohol	201
		2-Phenylethanol	201
	Carbonyls	2-Pentanone	201
		Hexanal	201
		(*E*)-2-Hexenal	201
		2,4-Dimethyl-3-pentan-one	201
		2-Methyl-3-hexanone	201
		Octanal	201
		4-Octanone	201
		Nonanal	201
		Decanal	201
		Geranylacetone	201
		Benzaldehydea	201
		β-Ionone	201
		Acetophenone	201
		4-Methoxybenzaldehyde	201
	Esters	Ethyl acetate	201
		Propylacetate	201
		Methyl 2-methylbutanoate	201
		Methyl dodecanoate	201
		Ethyl dodecanoate	201
		Methylethyl tetradecanoate	201
		Methyl (*E*)-cinnamate	201
		Ethyl ebcinnamate	201
		Dibutyl adipate	201
	Hydrocarbons	*o*-Xylene	201
		m-Xylene	201
		p-Xylene	201
		Ethylbenzene	201

		Naphthalene	201
		δ-3Carene	201
		p-Cymene	201
		Limonene	201
		(*E*)-Ocimene	201
		(*Z*)-Ocimene	201
	Carboxylic acids	Benzoic acid	201
		Cinnamic acid	201
	Miscellaneous compounds	δ-Octalactone	201
		N,N-Dimethyiformamide	201
		Dimethyldisulfide	201

References

1. Habermam, J. Über das Glycyrrhizin; erste Abhandlung. *Justus Liebig's Annalen der Chemie* **1879**, *197*, 105–125.
2. Sabbioni, C.; Mandrioli, R.; Ferranti, A.; Bugamelli, F.; Saracino, M.A.; Forti, G.C.; Fanali, S.; Raggi, M.A. Separation and analysis of glycyrrhizin, 18β-glycyrrhetic acid and 18α-glycyrrhetic acid in liquorice roots by means of capillary zone electrophoresis. *J. Chrom. A.* **2005**, *1081*, 65–71.
3. Shin, Y.W.; Bae, E.A.; Lee, B.; Lee, S.H.; Kim, J.A.; Kim, Y.S.; Kim, D.H. In vitro and in vivo antiallergic effects of *Glycyrrhiza glabra* and its components. *Planta Med*. **2007**, *73*, 257–261.
4. Shibano, M.; Ozaki, K.; Watanabe, H.; Tabata, A.; Taniguchi, M.; Baba, K. Determination of flavonoids in licorice using acid hydrolysis and reversed-phase hplc and evaluation of the chemical quality of cultivated licorice. *Planta Med.* **2010**, *76*, 729–733.
5. Farag, M.A.; Porzel, A.; Wessjohann, L.A. Comparative metabolite profiling and fingerprinting of medicinal licorice roots using a multiplex approach of GC-MS, LC-MS and 1D NMR techniques. *Phytochemistry* **2012**, *76*, 60–72.
6. Schmid, C.; Dawid, C.; Peters, V.; Hofmann, T. Saponins from European licorice roots (*Glycyrrhiza glabra*). *J. Nat. Prod.* **2018.**
7. Beaton, J.M.; Spring, F.S. Triterpenoids. Part LI. The Isolation and characterisation of glabric acid, a new triterpenoid acid from liquorice root. *J. Chem. Soc.* **1956**, 2417-2419.
8. Elgamal, M.H.A.; Fayez, M.B.E.; Snatzke, G. Constituents of local plants—VI. Liquoric acid, a new triterpenoid from the roots of *Glycyrrhiza glabra* L. *Tetrahedron* **1965**, *21*, 2109–2115.
9. Saitoh, T.; Shibata, S. Chemical studies on the oriental plant drugs. XXII. Some new constituents of licorice root. (2) glycyrol, 5-*O*-methylglycyrol and isoglycyrol. *Chem. Pharm. Bull*. **1969**, *17*, 739–734.
10. Beasley, T.H.; Sr.; Ziegler, H.W.; Bell, A.D. Separation of major components in licorice using high-performance liquid chromatography. *J. Chrom. A* **1979**, *175*, 350–355.
11. Amagaya S.; Sugishita E.; Ogihara, Y. Separation and quatitative analysis of 18α-glycyrrhetinic acid and 18β-glycyrrhetinic acid in Glycyrrhizae Radix by gas-liquid chromatogyaphy. *J. Chrom.* **1985**, *320*, 430–434.
12. Chamoli, A.; Ahmad, M.; Hasan, M.; Panda, B.P. Simultaneous determination of 18α-glycyrrhetinic acid and 18β-glycyrrhetinic acid in *Glycyrrhiza glabra* root by reversed phase high-performance liquid chromatography. *Drug Des. Devel. Ther.* **2016**, *7*, 59–62. 78.
13. Price, K.R.; Johnson, I.T.; Fenwick, G.R.; Malinow, M.R. The chemistry and biological significance of saponins in foods and feeding stuffs. *Crit. Rev. Food Sci. Nutr.* **1987**, *26*, 27–135.
14. Elgamal, M.H.A.; Hady, F.K.A.; Hanna, A.G.; Mahran, G.H.; Duddeck, H. A further contribution to the triterpenoid constituents of *Glycyrrhiza glabra* L. *Z. Naturforsch. C* **1990**, *45c*, 937–941.
15. Hu, J.; Shen, F. A survey of the studies on chemical conctituents cof Glyzyrrhiza. *Natural Products research and Development* **1996**, *8*, 77–91. (In Chinese)
16. Wei, J.; Zheng, Y.; Li, C.; Tang, Y.; Peng, G. Bioactive constituents of oleanane-type triterpene saponins from the roots of *Glycyrrhiza glabra*. *J. Asian Nat. Prod. Res.* **2014**, *16*, 1044–1053.
17. Rizzato, G.; Scalabrin, E.; Radaelli, M.; Capodaglio, G.; Piccolo, O. A new exploration of licorice metabolome. *Food Chem.* **2017**, *221*, 959–968.
18. Wei, J. Bioactive constituents of oleanane-type triterpene saponins from the roots of *Glycyrrhiza glabra*. M.D. Thesis, Nanjing University of Chinese Medicine, China, June 2015. (In Chinese)

19. Shaikh, A. An update on secondary metabolites from *Glycyrrhiza* species. *J. Basic App. Sci.* **2017**, *13*, 431-436.
20. Farag, M.A.; Wessjohann, L.A. Volatiles profiling in medicinal licorice roots using steam distillation and solid-phase microextraction (SPME) coupled to chemometrics. *J. Food Sci.* **2012**, *77*, 1179–1184.
21. Zhao, T.; Dong, Y.; Zhao, M. Gas chromatography-mass spectrometry analysis of volatiles components obtained from the *Glycyrrhiza glabra* L. leaf and root. *Science and Technology of Food Industry.* **2013**, *34*, 96–99. (In Chinese)
22. Kameoka, H.; Nakai, K. Components of essential oil from the root of *Glycyrrhiza glabra*. *Nippon Nageikagaku Kaishi*. **1987**, *61*, 1119–1121.
23. Reiners, W. 7-Hydroxy-4'-methoxy-isoflavon (formononetin) aus Süssholzwurzel. Über Inhaltsstoffe der Süssholzwurzel. II. *Speeialia* **1966**, *15*, 359.
24. Mitscher, L.A.; Park, Y.H.; Omoto, S.; Clark, G. W.; Clark, D. Antimicrobial agents from higher plants, *Glycyrrhiza glabra* L. (var. Spanish). I. Some antimicrobial isoflavans, isoflavenes, flavanones and isoflavones. *HeteroCycles* **1978**, *9*, 1533–1538.
25. Hayashi, H.; Hiraoka, N.; Ikeshiro, Y.; Yamamoto, H. Organ specific localization of flavonoids in *Glycyrrhiza glabra* L. *Plant Sci.* **1996**, *116*, 233–238.
26. Khalaf, I.; Vlase, L.; Lazăr, D.; Corciovă, A.; Ivãnescu, B.; Lazăr, M.I. HPLC-MS study of phytoestrogens from *Glycyrrhiza glabra*. *Farmacia* **2010**, *58*, 89–94.
27. Kattaev, N.Sh.; Nikonov, G.K. Flavonoids of *Glycyrrhiza glabra*. *Chem. Nat. Compd.* **1974**, *10*, 94–95.
28. Kinoshita, T.; Saitoh, T.; Shibata, S. The occurrence of an isoflavene and the corresponding isoflavone in licorice root. *Chem. Pharm. Bull*. **1976**, *24*, 991–994.
29. Kuroda, M.; Mimaki, Y.; Honda, S.; Tanaka, H.; Yokota, S.; Tatsumasa, M.T. Phenolics from *Glycyrrhiza glabra* roots and their PPAR-γ ligand-binding activity. *Bioorg. Med. Chem.* **2010**, *18*, 962–970.
30. Bhardwaj, D.K.; Murari, R.; Seswadri, T.R.; Singh, R. Glyzarin, a new isoflavone from *Glycyrrhiza glabra*. *Phymckeminry* **1977**, *16*, 402 –403.
31. Kinoshita, T.; Saitoh, T.; Shibata, S. A new isoflavone from licorice root. *Chem. Pharm. Bull.* **1978**, *26*, 141–143.
32. Hatano, T.; Kagawa, H.; Yasuhara, T.; Okuda, T. Two new flavonoids and other constituents in licorice root: their relative astringency and radical scavenging effects. *Chem. Pharm. Bull*. **1988**, *36*, 2090–2097.
33. Hatano, T.; Yasuhara, T.; Fukuda, T.; Noro, T.; Okuda, T. Phenolic constituents of licorice. II. Structures of licopyranocoumarin, licoarylcoumarin and glisoflavone, and inhibitory effects of licorice phenolics on xanthine oxidase. *Chem. Pharm. Bull.* **1989**, *37*, 3005–3009.
34. Fukai, T.; Tantai, L.; Nomura, T. Isoprenoid-substituted flavonoids from *Glycyrrhiza glabra*. *Phytochemistry* **1996**, *43*, 531–532.
35. Fukai, T.; Sheng, C.; Horikoshi, T.; Nomura, T. Isoprenylated flavonoids from underground parts of *Glycyrrhiza glabra*. *Phytochemistry* **1996**, *43*, 1119–1124.
36. Bhardwaj, D.K.; Singh, R. 'Glyzaglabrin', a new isoflavone from *Glycyrrhiza glabra. Letters to the Editor.* **1977**, *46*, 753.
37. Kinoshita, T.; Tamura, Y.; Mizutani, K. The isolation and structure elucidation of minor isoflavonoids from licorice of *Glycyrrhiza glabra* origin. *Chem. Pharm. Bull.* **2005**, *53*, 847–849.
38. Zhang, Y.; Yang, Y.; Gong, H.; Zhu, H. A systematic review of the comparison of three medicinal licorices, based on differences of the types and contents about their bioactive components. *J. Chem. Biol. Pharm. Chem*. **2018**, *1*, 1.
39. Bhardwaj, D.K.; Murari, R.; Seswadri, T.R.; Singh, R. Occurrence of 2-methylisoflavones in *Glycyrrhiza glabra*. *Phetochmistry* **1976**, *15*, 352–353.
40. Yang, L.; Liu, Y.; Lin, S. HPLC analysis of flavonoids in the root of six *Glycyrrhiza* species. *Acta Pharm. Sin.* **1990**, *25*, 840–848. (In Chinese)
41. Saitoh, T.; Kinoshita, T.; Shibata, S. New isoflavan and flavanone from licorice root. *Chem. Pham. Bull*. **1976**, *24*, 752–755.
42. Kinoshita, T.; Kajiyama, K.; Hiraga, Y.; Takahashi, K.; Tamura, Y.; Mizutani, K. Isoflavan derivatives from *Glycyrrhiza glabra* (licorice). *HeteroCycles* **1996**, *43*, 581–588.
43. Fukai, T.; Satoh, K.; Nomura, T.; Sakagamic, H. Preliminary evaluation of antinephritis and radical scavenging activities of glabridin from *Glycyrrhiza glabra*. *Fitoterapia* **2003**, *74*, 624–629.
44. Kamal, Y.T.; Singh, M.; Tamboli, E.T.; Parveen, R.; Zaidi, S.M.A.; Ahmad, S. Rapid RP-HPLC method for the quantification of glabridin in crude drug and in polyherbal formulation. *J. Chromatogr. Sci.* **2012**, *50*, 779–784.

45. Li, K.; Ji, S.; Song, W.; Kuang, Y.; Lin, Y.; Tang, S.; Cui, Z.; Qiao, X.; Yu, S.; Ye, M. Glycybridins A−K, bioactive phenolic compounds from *Glycyrrhiza glabra*. *J. Nat. Prod.* **2017**, *80*, 334–346.
46. Shibata, S.; Saitoh, T. The chemical studies on the oriental plant drugs. XIX. Some new constituents of licorice root. (1). The structure of licoricidin. *Chem. Pharm. Bull.* **1968**, *16*, 1932–1936.
47. Fukai, T.; Cai, B.; Maruno, K.; Miyakawa, Y.; Konishi, M.; Nomura, T. An isoprenylated flavanone from *Glycyrrhiza glabra* and rec-assay of licorice phenols. *Phytochemistry* **1998**, *49*, 2005–2013.
48. Fukai, T.; Nishizawa, J.; Yokoyama, M.; Tantai, L.; Nomura, T. Five new isoprenoid-substituted flavonoids, kanzonols M-P and R, from two *Glycyrrhiza* species. *HeteroCycles* **1994**, *38*, 1089–1098.
49. Saitoh, T.; Shibata, S. New type chalcones from licorice root. *Tetrahedron Lett.* **1975**, 50, 4461–4462.
50. Zhuo, Y.; Wang, X.; Chen, W. Determination of licochalcone A in *Glycyrrhiza glabra* by HPLC. *Chinese Journal of Hospital Pharmacy* **2009**, *29*, 2134–2135. (In Chinese)
51. Fenwick, G.R.; Lutomski, J.; Nieman, C. Liquorice, *Glycyrrhiza glabra* L. - composition, uses and analysis. *Food Chem.* **1990**, *38*, 119–143.
52. Kinoshita, T.; Kajiyama, K.; Hiraga, Y.; Takahashi, K.; Tamura, Y.; Mizutani, K. The isolation of new pyrano-2-arylbenzofuran derivatives from the root of *Glycyrrhiza glabra*. *Chem. Pharm. Bull.* **1996**, *44*, 1218–1221.
53. Rafi, M.M.; Vastano, B.C.; Zhu, N.; Ho, C.; Ghai, G.; Rosen, R.T.; Gallo, M.A.; Dipaola, R.S. Novel polyphenol molecule isolated from licorice root (*Glycrrhiza glabra*) induces apoptosis, G2/M cell cycle arrest, and Bcl-2 phosphorylation in tumor cell lines. *J. Agric. Food Chem.* **2002**, *50*, 677–684.
54. Wu, Y.; Meng, X.; Bao, Y.; Wang, S.; Kang, T. Simultaneous quantitative determination of nine active chemical compositions in traditional Chinese medicine *Glycyrrhiza* by RP-HPLC with full-time five-wavelength fusion method. *Am. J. Chin. Med.* **2013**, *41*, 211–219.
55. Khan, N.; Ali, S.A. HRLC-MS analysis of isoliquiritigenin from the root extract of *Glycyrrhiza glabra* for developing a novel depigmenting agent. *Bioscience Biotechnology Research Communications* **2014**, *7*, 89–93.
56. Gaur, R.; Gupta, V.K.; Singh, P.; Pal, A.; Darokar, M.P.; Bhakuni, R.S. Drug resistance reversal potential of isoliquiritigenin and liquiritigenin isolated from *Glycyrrhiza glabra* against methicillin-resistant Staphylococcus aureus (MRSA). *Phytother. Res.* **2016**, *30*, 1708–1715. 122.
57. Yang, R.; Yuan, B.; Ma, Y.; Zhou, S.; Zhang, H.; Liu, J.; Li, W.; Liu, Y. Simultaneous determination of liquiritin, isoliquiritin, liquiritigenin and isoliquiritigenin in *Glycyrrhiza uralensis* Fisch., *Glycyrrhiza glabra* L., and *Glycyrrhiza inflata* Bat. by HPLC. *Chinese Journal of Pharmaceutical Analysis* **2016**, *36*, 1729–1736.
58. Paris, R.; Guillot, M. Liquiritoside, flavonoside from root of licorice, *Glycyrrhiza glabra* L. Annales Pharmaceutiques Françaises. **1955**, *13*, 592–595.
59. Kaur, R.; Kaur, H.; Dhindsa, A.S. *Glycyrrhiza glabra*: a phytopharmacological review. *Int. J. Pharm. Sci. Res.* **2013**, *4*, 2470-2477.
60. Fukai, T.; Wang, Q.; Kitaqawa, T.; Kusano, K.; Nomura, T.; Iitaka, Y. Structures of six isoprenoio-substiwted flavonoids, gancaonins F, G, R, I, glycyrol, and isoglycyrol from xibei licorice (*Glycyrrhiza* sp.). *HeteroCycles* **1989**, *29*, 1761–1772.
61. Demizu, S.; Kajiyama, K.; Takahashi, K.; Hiraga, Y.; Yamamoto, S.; Tamura, Y.; Okada, K.; Kinoshita, T. Antioxidant and antimicrobial constituents of licorice: isolation and structure elucidation of a new benzofuran derivative. *Chem. Pharm. Bull.* **1988**, *36*, 3474–3479.
62. Saitoh, T.; Kinoshita, T.; Shibata, S. Flavonols of licorice root. *Chem. Pharm. Bull.* **1976a**, *24*, 1242–1245.
63. Asl, M.N.; Hosseinzadeh, H. Review of pharmacological effects of *Glycyrrhiza* sp. and its bioactive compounds. *Phytother. Res.* **2008**, *22*, 709–724.
64. Li, J.; Wang, Y.; Deng, Z. Two new compounds from *Glycyrrhiza glabra*. *J. Asian Nat. Prod. Res.* **2005**, *7*, 677–680.
65. Dirican, E.; Turkez, H. In vitro studies on protective effect of *Glycyrrhiza glabra* root extracts against cadmium-induced genetic and oxidative damage in human lymphocytes. *Cytotechnology* **2014**, *66*, 9–16.
66. Wang, X. Determination of the content of liquiritin in licorice by thin layer densitometry. *Chinese Journal of Pharmaceutical Analysis* **1990**, *10*, 351–352. (In Chinese)
67. Dong, Y. Purification of flavonoids from *Glycyrrhiza glabra* L. leaf and their biological activities and applications. **2016**.
68. Reiners, W. Cumarine und Hydroxyzimtsäuren aus Süßholzwurzel. *Naturwissenschaften* **1964**, *51*, 193.

69. Frattini, C.; Bicchi, C.; Barettini, C.; Nano, G.M. Volatile flavor components of licorice. *J. Agr. Food Chem.* **1977**, *25*. 1238–1241.
70. Bhardwaj, D.K.; Murari, R.; Seswadri, T.R.; Singh, R. Liqcoumarin, a novel coumarin from *Glycyrrhiza glabra*. *Phytochemistry* **1976**, *15*, 1182–1183.
71. Kinoshita, T.; Saitoh, T.; Shibata, S. A new 3-arylcoumarin from licorice root. *Chem. Pharm. Bull.* **1978a**, *26*, 135–140.
72. Ma, J. Studies on the secondary metabolites of four *Glycyrrhiza* species in China. M.D. Thesis, Northwest Normal University, Gansu, China, May 2004. (In Chinese)
73. Näf, R.; Jaquier, A. New lactones in liquorice (*Glycyrrhiza glabra* L.). *Flavour. Fragr. J.* **2006**, *21*, 193–197.
74. Biondi, D.M.; Rocco, C.; Ruberto, G. New dihydrostilbene derivatives from the leaves of *Glycyrrhiza glabra* and evaluation of their antioxidant activity. *J. Nat. Prod.* **2003**, *66*, 477–480.
75. Biondi, D.M.; Rocco, C.; Ruberto, G. Dihydrostilbene derivatives from *Glycyrrhiza glabra* leaves. *J. Nat. Prod.* **2005**, *68*, 1099–1102.
76. Hayashi, H.; Yasuma, M.; Hiraoka, N.; Ikeshiro, Y.; Yamamoto, H.; Yeşilada, E.; Sezik, E.; Honda, G.; Tabat, M. Flavonoid variation in the leaves of *Glycyrrhiza glabra*. *Phytochemistry* **1996**, *42*, 701–704.
77. Hayashi, H.; Hiraoka, N.; Ikeshiro, Y.; Yamamoto, H.; Yoshikawa, T. Seasonal variation of glycyrrhizin and isoliquiritigenin glycosides in the root of *Glycyrrhiza glabra* L. *Biol. Pharm. Bull.* **1998**, *21*, 987–989.
78. Statti, G.A.; Tundis, R.; Sacchetti, G.; Muzzoli, M.; Bianchi, A.; Menichini, F. Variability in the content of active constituents and biological activity of *Glycyrrhiza glabra. Fitoterapia* **2004**, *75*, 371–374.
79. Chin, Y.; Jung, H.; Liu, Y.; Su, B.; Castoro, J.A.; Keller, W.J.; Pereira, M.A.; Kinghorn, A.D. Anti-oxidant constituents of the roots and stolons of licorice (*Glycyrrhiza glabra*). *J. Agr. Food Chem.* **2007**, *55*, 4691–4697.
80. Rathee, P.; Rathee, S.; Ahuja, D. Simultaneous quantification of glycyrrhetinic acid and apigenin using HPTLC from *Glycyrrhiza glabra* Linn. *Eurasian J. Ana. Chem.* **2010**, *5*, 95–103.
81. Li, G.; Nikolic, D.; Breemen, R.B.V. Identification and chemical standardization of licorice raw materials and dietary supplements using UHPLC-MS/MS. *J. Agr. Food Chem.* **2016**, *64*, 8062–8070.
82. Zeng, L.; Lou, Z.; Zhang, R. Quality evaluation of Chinese licorice. *Acta Pharma Sin*. **1991**, *26*, 788–793. (In Chinese)
83. Hatano, T.; Fukuda, T.; Liu, Y.; Noro, T.; Okuda, T. Phenolic constituents of licorice. IV. Correlation of phenolic constituents and licorice specimens from various sources, and inhibitory effects of licorice extracts on xanthine oxidase and monoamine oxidase. *Yakugaku Zasshi* **1991**, *111*, 311–321.
84. Ma, S.; Abulizi, M. Kahaer, B.; He, Q. Study on the preparation technology of isoflavon glabridin from *Glycyrrhiza glabra* L. *Journal of Xinjiang Medical University* **2007**, *30*, 692–694. (In Chinese)
85. Li, J.; Song, X.; Yu, B.; Li, Y. Content determination of glabridin in *Glycyrrhiza glabra* L. with HPLC. *Tianjin Journal of Traditional Chinese Medicine* **2008**, *25*, 157–158. (In Chinese)
86. Luo, C.; Wang, X.; Jiang, F. Determination of glabridin in *Glycyrrhiza glabra* L. by RP-HPLC. *Journal of Nongken Medicine* **2009**, *31*, 311–313.
87. Ammar, N.M.; El-Hawary, S.S.; El-anssary, A.A.; Othman, N.; Galal, M.; El-Desoky, A.H. Phytochemical and clinical studies of the bioactive extract of *Glycyrrhiza glabra* L. family leguminosae. *International Journal of Phytomedicine* **2012**, *4*, 429–436.
88. Martins, N.; Barros, L.; Dueñas, M.; Santos-Buelga, C.; Ferreira, I.C.F.R. Characterization of phenolic compounds and antioxidant properties of *Glycyrrhiza glabra* L. rhizomes and roots. *RSC Adv.* **2015**, *5*, 26991–26997.
89. Yu, J.; Wang, S.; Gao, W. Identification of vitexin in *Glycyrrhiza glybra* L. *Strait Pharmaceutical Journal* **2012**, *24*, 40–41. (In Chinese)
90. Hayashi, H.; Fukui, H.; Tabata, M. Examination of triterpenoids produced by callus and cell suspension cultures of *Glycyrrhiza glabra*. *Plant Cell Rep.* **1988**, *7*, 508–511.
91. Asada, Y.; Li, W.; Yoshikawa, T. The first prenylated biaurone, licoagrone from hairy root cultures of *Glycyrrhiza glabra*. *Phytochemistry* **1999**, *49*, 1015–1019.
92. Li, W.; Asada, Y.; Yoshikawa, T. Antimicrobial flavonoids from *Glycyrrhiza glabra* hairy root cultures. *Planta Med*. **1998**, *64*, 746–747.
93. Asada, Y.; Li, W.; Yoshikawa, T. Isoprenylated flavonoids from hairy root cultures of *Glycyrrhiza glabra*. *Phytochemistry* **1998**, *47*, 389–392.

94. Hatano, T.; Fukuda, T.; Miyase, T.; Noro, T.; Okuda, T. Phenolic constituents of licorice. III. Structures of glicoricone and licofuranone, and inhibitory effects of licorice constituents on monoamine oxidase. *Chem. Pharm. Bull.* **1991b**, *39*, 1238–1243.
95. Rahman, H.; Khan, I.; Hussain, A.; Shahat, A.A.; Tawab, A.; Qasim, M.; Adnan, M.; Al-Said, M.S.; Ullah, R.; Khan, S.N. *Glycyrrhiza glabra* HPLC fractions: identification of aldehydo isoophiopogonone and liquirtigenin having activity against multidrug resistant Bacteria. *BMC Complement.Altern. Med.* **2018**, *18*.
96. Murakami, N.; Saka, M.; Shimada, H.; Matsuda, H.; Yamahara, J.; Yoshikawa, M. New bioactive monoterpene glycosides from Paeoniae Radix. *Chem. Pharm. Bull*. **1996**, *44*, 1279–1281.
97. Wang, X.; Jiao, W.; Liao, X.; Peng, S.; Ding, L. Monoterpene glycosides from the roots of *Paeonia lactiflora*. *Chinese Chemical Letters* **2006**, *17*, 916–918.
98. Zhang, X.; Gao, C.; Wang, J.; Li, X. A new monoterpene glycoside from *Paeonia lactiflora* Pall. *Acta Pharm. Sin*. **2002**, *37*, 705–708. (In Chinese)
99. Wang, Q.; Guo, H.; Huo, C.; Shi, Q.; Ye, M.; Bi, K.; Guo, D. Chemical constituents in root of *Paeonia lactiflora*. *Chinese Traditional and Herbal Drugs* **2007**, *38*, 972–976. (In Chinese)
100. Yen, P.; Kiem, P.; Nhiem, N.; Tung, N.; Quang, T.; Minh, C.; Kim, J.; Choi, E. A new monoterpene glycoside from the roots of *Paeonia lactiflora* increases the differentiation of osteoblastic MC3T3-E1 cells. *Arch. Pharm. Res.* **2007**, *30*, 1179–1185.
101. Kim, S.H.; Lee, M.K.; Lee, K.Y.; Sung, S.H.; Kim, J.; Kim, Y.C. Chemical constituents isolated from *Paeonia lactiflora* roots and their neuroprotective activity against oxidative stress in vitro. *J. Enzyme Inhib. Med. Chem*. **2009**, *24*, 1138–1140.
102. Jian, Z.; Yu, J.; Wang, W. RP-HPLC determination of main chemical components in different parts and different harvest periods of *Paeonia lactiflora*. *Acta Pharm. Sin.* **2010**, *45*, 489–493. (In Chinese)
103. Tan, J.; Zhao, Q.; Yang, L.; Shang, Z.; Du, Z.; Yan, M. Chemical constituents in roots of *Paeonia lactiflora*. *Chinese Traditional and Herbal Drugs* **2010**, *41*, 1245–1248. (In Chinese)
104. He, X.; Han, L.; Huang, X. A new phenolic glucoside from *Paeonia lactiflora*. *Chinese Herbal Medicines* **2011**, *3*, 84–86.
105. Li, P.; Zhang, Z.; Li, T.; Zhang, Y.; Sze, S.C.; Wang, G.; Li, Y.; Ye, W. Monoterpene derivatives from the roots of *Paeonia lactiflora* and their anti-proliferative activity. *Fitoterapia* **2014**, *98*, 124–129.
106. Bae, J.; Kim, C.Y.; Kim, H.J.; Park, J.H.; Ahn, M. Differences in the chemical profiles and biological activities of *Paeonia lactiflora* and *Paeonia obovata*. *J. Med. Food* **2015**, *18*, 224–232.
107. Liu, Y.; Ma, Y.; Yang, B.; Xiao, J.; Long, F.; Xu, Z.; Lv, G. Determination of bioactive components in *Paeonia lactiflora* roots cultivated in various areas by UHPLC. *Journal of Chinese Medicinal Materials* **2016**, *39*, 980–985. (In Chinese)
108. Shi, Y. Chemical constituents with anti-allergic activity from red peony root and a horticultural cultivar of *Paeonia lactiflora* and monoterpenoids profiles of peony related species. Ph.D. Thesis, University of Toyama, Yoyama, Japan, March 2016.
109. Liu, P.; Xu, Y.; Yan, H.; Chen, J.; Shang, E.; Qian, D.; Jiang, S.; Duan, J. Characterization of molecular signature of the roots of *Paeonia lactiflora* during growth. *Chin. J. Nat. Med.* **2017**, *15*, 0785–0793.
110. Li, B.; Bhandari, D.R.; Römpp, A.; Spengler, B. High-resolution MALDI mass spectrometry imaging of gallotannins and monoterpene glucosides in the root of *Paeonia lactiflora*. *Sci. Rep.* **2016**, *6*.
111. Braca, A.; Kiem, P.V.; Yen, P.H.; Nhiem, N.X.; Quang, T.H.; Cuong, N.X.; Minh, C.V. New monoterpene glycosides from *Paeonia lactiflora*. *Fitoterapia* **2008**, *79*, 117–120.
112. Kim, J.S.; Yean, M.H.; Lee, J.Y.; Kim, Y.J.; Lee, E.J.; Lee, S.Y.; Kang, S.S. A new monoterpene glucoside from the roots of *Paeonia lactiflora*. *Helvetica Chimica Acta* **2008**, *91*, 85–89.
113. Ren, M.; Zhang, X.; Ding, R.; Dai, Y.; Tu, F.; Cheng, Y.; Yao, X. Two new monoterpene glucosides from *Paeonia lactiflora* Pall. *J. Asian Nat. Prod. Res.* **2009**, *11*, 670–674.
114. Washida, K.; Yamagaki, T.; Iwashita, T.; Nomoto, K. Two new galloylated monoterpene glycosides, 4-O-galloylalbiflorin and 4'-O-galloylpaeoniflorin, from the roots of *Paeonia lactiflora* (Paeoniae Radix) grown and processed in Nara Prefecture, Japan. *Chem. Pharm. Bull.* **2009**, *57*, 1150–1152.
115. Wang, H.; Gu, W.; Chu, W.; Zhang, S.; Tang, X.; Qin, G. Monoterpene glucosides from *Paeonia lactiflora. J. Nat. Prod.* **2009**, *72*, 1321–1324.

116. Fu, Q.; Wang, S.; Zhao, S.; Chen, X.; Tu, P. Three new monoterpene glycosides from the roots of *Paeonia lactiflora*. *J. Asian Nat. Prod. Res.* **2013**, *15*, 697–702.
117. Fu, Q.; Yu, T.; Yuan, H.; Song, Y.; Zou, L. Paeonidanins F-H: three new dimeric monoterpene glycosides from *Paeonia lactiflora* and their anti-inflammatory activity. *Phytochem. Lett.* **2015**, *13*, 386–389.
118. Parker, S.; May, B.; Zhang, C.; Zhang, A.L.; Lu, C.; Xue, C.C. A pharmacological review of bioactive constituents of *Paeonia lactiflora* Pallas and *Paeonia veitchii* Lynch. *Phytother. Res*. **2016**, *30*, 1445–1473.
119. Fu, Q.; Qiu, L.; Yuan, H.; Yu, T.; Zou, L. Paeonenoides D and E: two new nortriterpenoids from *Paeonia lactiflora* and their inhibitory activities on NO production. *Helvetica Chimica Acta* **2016**, *99*, 46–49.
120. Fu, F.; Shang, T.; Hsu, T. Studies on the chemical constituents of the Chinese medical drug, root of *Paeonia lactiflora* Pall. *Yao Xue Xue Bao* **1963**, *10*, 555–557.
121. Liu, W.; Li, D.; Yang, H.; Chen, Y.; Wei, J.; Kang, W.; Guo, X. Determination of oleanic acid and paeoniflorin in *Paeonia lactiflora* by ultrasound-assisted ionic liquid-reversed phase liquid chromatography. *Zhongguo Zhong Yao Za Zhi* **2015**, *40*, 443–449. (In Chinese)
122. Guo, D.; Ye, G.; Guo, H. A new phenolic glycoside from *Paeonia lactiflora*. *Fitoterapia* **2006**, *77*, 613–614.
123. He, C.; Peng, Y.; Zhang, Y.; Xu, L.; Gu, J.; Xiao, P. Phytochemical and biological studies of Paeoniaceae. *Chem. Biodivers.* **2010**, *7*, 805–838.
124. Ngan. L.T.M.; Jang, M.J.; Kwon, M.J.; Ahn, Y.J. Antiviral activity and possible mechanism of action of constituents Identified in *Paeonia lactiflora* root toward human rhinoviruses. *PLoS ONE* **2015**, *10.*
125. Stavri, M.; Mathew, K.T.; Bucar, F.; Gibbons, S. Pangelin, an antimycobacterial coumarin from Ducrosia anethifolia. *Planta Med.* **2003**, *69*, 956–959.
126. Shu, X.; Duan, W.; Liu, W.; Geng, Y.; Wang, X.; Yang, B.; Yang, P. Chemical constituents from flowers of *Paeonia lactiflora*. *Journal of Chinese Medicinal Materials* **2014**, *37*, 66–69. (In Chinese)
127. Magid, A.A.; Schmitt, M.; Prin, P.; Pasquier, L.; Voutquenne-Nazabadioko, L. In Vitro tyrosinase inhibitory and antioxidant activities of extracts and constituents of *Paeonia lactiflora* Pall. flowers. *The Natural Products Journal* **2017**, *7*, 237–245.
128. Shu, X.; Duan, W.; Liu, F.; Shi, X.; Geng, Y.; Wang, X.; Yang, B. Preparative separation of polyphenols from the flowers of *Paeonia lactiflora* Pall. by high-speed counter-current chromatography. *J. Chromatogr. B Analyt. Technol. Biomed. Life Sci.* **2014**, *947–948*, 62–67.
129. Tanaka, T.; Fukumori, M.; Ochi, T.; Kouno, I. Paeonianins A-E, new dimeric and monomeric ellagitannins from the fruits of *Paeonia lactiflora*. *J. Nat. Prod*. **2003**, *66*, 759–763.
130. Zhou, C.; Zhang, Y.; Sheng, Y.; Zhao, D.; Lv, S.; Hu, Y.; Tao, J. Herbaceous peony (*Paeonia lactiflora* Pall.) as an alternative source of oleanolic and ursolic acids. *Int. J. Mol. Sci.* **2011**, *12*, 655–667.
131. Liu, P.; Xu, Y.; Gao, X.; Zhu, X.; Du, M.; Wang, Y.; Deng, R.; Gao, J. Optimization of ultrasonic-assisted extraction of oil from the seed kernels and isolation of monoterpene glycosides from the oil residue of *Paeonia lactiflora* Pall. *Ind. Crop. Prod.* **2017**, *107*, 260–270.
132. Choi, C.W.; Choi, Y.H.; Cha, M.R.; Park, J.H.; Kim, Y.S.; Kim, Y.K.; Choi, S.U.; Yon, G.H.; Hong, K.S.; Kim, Y.H.; Ryu, S.Y. α-Glucosidase inhibitiors from seed extract of *Paeonia lactiflora*. *J. Korean Soc. Appl. Biol. Chem*. **2009**, *52*, 638–642.
133. Kim, H.J.; Chang, E.J.; Cho, S.H.; Chung, S.K.; Park, H.D.; Choi, S.W. Antioxidative activity of resveratrol and its derivatives isolated from seeds of *Paeonia lactiflora*. *Biosci. Biotechnol. Biochem.* **2002**, *66*, 1990–1993.
134. Yuk, H.J.; Ryu, H.W.; Jeong, S.H.; Curtis-Long, M.J.; Kim, H.J.; Wang, Y.; Song, Y.H.; Park, K.H. Profiling of neuraminidase inhibitory polyphenols from the seeds of *Paeonia lactiflora*. *Food Chem. Toxicol.* **2013**, *55*, 144–149.
135. Wu, S.; Luo, X.; Ma, Y.; Hao, X.; Wu, D. A new monoterpene glycoside from *Paeonia veitchii*. *Chinese Chemical Letters* **2002**, *13*, 430–431.
136. Wang, Q.; Liu, R.; Guo, H.; Ye, M.; Huo, C.; Bi, K.; Guo, D. Simultaneous LC determination of major constituents in red and white peony root. *Chromatographia* **2005**, *62*, 581–588.
137. Wu, S.; Chen, Y.; Yang, L.; Li, S.; Li, Z. Chemical constituents of *Paeonia veitchii*. *Chinese Traditional and Herbal Drugs* **2008**, *39*, 13–15. (In Chinese)
138. Xu, X.; Wu, Y. Studies on the separation of monoterpene glycosides from *Paeonia veitchii* Lynch. herbs. *Pharm. Chem. J.* **2016**, *50*, 568–572.

139. Fu, Q.; Tan, M.; Yuan, H.; Chen, J.; Fu, J. Monoterpene glycosides from *Paeonia veitchii*. *J. Asian Nat. Prod. Res.* **2017**, *19*, 22–27.
140. Wang, R.; Chou, G.; Zhu, E.; Wang, Z.; Bi, K. Studies on chemical constituents of *Paeonia veitchii* L. *Chinese Pharmaceutical Journal* **2007**, *42*, 662–663. (In Chinese)
141. Liang, W.; Ma, Y.; Geng, C.; Huang, X.; Xu, H.; Zhang, X.; Chen, J. Paeoveitols A-E from *Paeonia veitchii. Fitoterapia* **2015**, *106*, 36–40.
142. Wu, S.; Yang, S.; Wu, D.; Cheng, Y.; Peng, Q. Three novel 24,30-dinortriterpenoids, paeoneoides A-C, from *Paeonia veitchii*. *Helvetica Chimica Acta* **2005**, *88*, 259–265.
143. Liang, W.; Geng, C.; Zhang, X.; Chen, H.; Yang, C.; Rong, G.; Zhao, Y.; Xu, H.; Wang, H.; Zhou, N.; Ma, Y.; Huang, X.; Chen, J. (±)-Paeoveitol, a pair of new norditerpene enantiomers from *Paeonia veitchii*. *Org. Lett.* **2014**, *16*, 424–427.
144. Wang, R.; Chou, G.; Zhu, E.; Wang, Z.; Bi, K. A new phenolic glycoside from the roots of *Paeonia veitchii. J. Asian Nat. Prod. Res*. **2006**, *8*, 277–280.
145. Jia, N.; Shu, Q.; Wang, D.; Wang, L.; Liu, Z.; Ren, H.; Xu, Y.; Tian, D.; Tilt, K.M. Identification and characterization of anthocyanins by high-performance liquid chromatography–electrospray ionization–mass spectrometry in herbaceous peony species. *J. Am. Soc. for Hortic. Sci.* **2008**, *133*, 418–426.
146. Shimizu, M.; Fukumura, H.; Tsuji, H.; Tanaami, S.; Hayashi, T.; Morita, N. Anti-inflammatory constituents of topically applied crude drugs. I. Constituents and anti-inflammatory effect of *Eriobotrya japonica* Lindl. *Chem. Pharm. Bull.* **1986**, *34*, 2614–2617.
147. Liang, Z.Z.; Aquino, R.; Feo, V.D.; Simone, F.D.; Pizza, C. Polyhydroxylated triterpenes from *Eriobotrya japonica*. *Planta Med*. **1990**, *56*, 330–332.
148. Jung, H.A.; Park, J.C.; Chung, H.Y.; Kim, J.; Choi, J.S. Antioxidant flavonoids and chlorogenic acid from the leaves of *Eriobotrya japonica. Arch. Pharm Res.* **1999**, *22*, 213–218.
149. Ju, J.; Zhou, L.; Lin, G.; Liu, D.; Wang, L.; Yang, J. Studies on constituents of triterpene acids from *Eriobotrya japonica* and their anti-inflammatory and antitussive effects. *Chinese Pharmaceutical Journal* **2003**, *8*, 753–757. (In Chinese)
150. Banno, N.; Akihisa, T.; Tokuda, H.; Yasukawa, K.; Taguchi, Y.; Akazawa, H.; Ukiya, M.; Kimura, Y.; Suzuki, T.; Nishino, H. Anti-inflammatory and antitumor-promoting effects of the triterpene acids from the leaves of *Eriobotrya japonica. Biol. Pharm. Bull*. **2005**, *28*, 1995–1999.
151. Hong, Y.; Lin, S.; Huang, X. Determination of ursolic acid in *Eriobotrya* leaves and terpenoid fingerprinting. *Acta Hortic* **2007**, *750*, 225–232.
152. Lv, H.; Chen, J.; Li, W.; Zhang, H. Studies on the Triterpenes from loquat leaf (*Eriobotrya japonica*). *Journal of Chinese Medicinal Materia* **2008**, *31*, 1351–1354. (In Chinese)
153. Li, E.; Luo, J.; Kong, L. Qualitative and quantitative determination of seven triterpene acids in *Eriobotrya japonica* Lindl. by high-performance liquid chromatography with photodiode array detection and mass spectrometry. *Phytochem. Anal.* **2009**, *20*, 338–343.
154. Rollinger, J.M.; Kratschmar, D.V.; Schuster, D.; Pfisterer, P.H.; Gumy, C.; Aubry, E.M.; Brandstötter, S.; Stuppner, H.; Wolber, G.; Odermatt, A. 11β-Hydroxysteroid dehydrogenase 1 inhibiting constituents from *Eriobotrya japonica* revealed by bioactivity-guided isolation and computational approaches. *Bioorganic. Med. Chem.* **2010**, *18*, 1507–1515.
155. Yang, Y.; Huang, Y.; Huang, C.; Lv, X.; Liu, L.; Wang, Y.; Li, J. Antifibrosis effects of triterpene acids of *Eriobotrya japonica* (Thunb.) Lindl. leaf in a rat model of bleomycin-induced pulmonary fibrosis. *J. Pharm. Pharmacol.* **2012**, *64*, 1751–1760.
156. Wu, Q.; Wang, M.; Simon, J.E.; Yu, S.; Xiao, P.; Ho, C. Studies on the chemical constituents of loquat leaves (*Eriobotrya japonica*). *ACS Symposium Series.* **2003**, *859*, 292–306.
157. Kikuchi, T.; Akazawa, H.; Tabata, K.; Manosroi, A.; Manosroi, J.; Suzuki, T. Akihisa, T. 3-*O*-(*E*)-*p*-coumaroyl tormentic acid from *Eriobotrya japonica* leaves induces caspase-dependent apoptotic cell death in human leukemia cell line. *Chem. Pharm. Bull*. **2011**, *59*, 378–381.
158. Li, E.; Zhou, G.; Kong, L. Chemical constituents from the leaves of *Eriobotrya japonica*. *Chin. J. Nat. Med.* **2009**, *7*, 190–192.
159. De Tommasi, N.; De Simone, F.; Cirino, G.; Cicala, C.; Pizza C. Hypoglycemic effects of sesquiterpene glycosides and polyhydroxylated triterpenoids of *Eriobotrya japonica*. *Planta Med.* **1991**, *57*, 414–416.

160. Hu, C.; Chen, L.; Xin, Y.; Cai, Q. Determination of corosolic acid in *Eriobotrya japonica* leaves by reversed-phase high performance liquid chromatography. *Chinese Journal of Chromatography* **2006**, *24*, 492–494. (In Chinese)
161. Matalka, K.Z.; Abdulridha, N.A.; Badr, M.M.; Mansoor, K.; Qinna, N.A.; Qadan, F. *Eriobotrya japonica* water extract characterization: an inducer of interferon-gamma production mainly by the JAK-STAT pathway. *Molecules* **2016**, *21*.
162. Ito, H.; Kobayashi, E.; Li, S.; Hatano, T.; Sugita, D.; Kubo, N.; Shimura, S.; Itoh, Y.; Yoshida, T. Megastigmane glycosides and an acylated triterpenoid from *Eriobotrya japonica*. *J. Nat. Prod.* **2001**, *64*, 737–740.
163. De Tommasi, N.; De Simone, F.; Pizza, C.; Mahmood, N.; Moore, P.S.; Conti, C.; Orsi, N.; Stein, M.L. Constituents of *Eriobotrya japonica*. A study of their antiviral properties. *J. Nat. Prod*. **1992**, *55*, 1067–1073.
164. Shimizu, M.; Uemitsu, N.; hirota, M.; atsumoto, K.; Tezuka, Y. A new triterpene ester from *Eriobotrya japonica. Chem. Pharm. Bull*. **1996**, *44*, 2181–2182.
165. Chen, J.; Li, W.; Wu, J.; Ren, B.; Zhang, H. Chemical constituents of *Eriobotrya japonica* leaf. *Journal of Plant Resources and Environment* **2006**, *15*, 67–68.
166. Hong, Y.; Huang, S.; Wu, J.; Lin, S. Identification of essential oils from the leaves of 11 species of *Eriobotrya*. *Pakistan Journal of Botany* **2010**, *42*, 4379–4386.
167. Yanagisawa, H.; Ohshima, Y.; kada, Y.; akahashi, K.; hibata, S. A sesquiterpene glycoside, loquatifolin A, from the leaves of *Eriobotrya japonica*. *Chem. Pharm. Bull.* **1988**, *36*, 1270–1274.
168. De Tommasi, N.; De Simone, F.; Aquino, R.; Pizza, C. Plant metabolites. New sesquiterpene glycosides from *Eriobotrya japonica*. *J. Nat. Prod*. **1990**, *53*, 810–815.
169. De Tommasi, N.; Aquino, R.; De Simone, F.; Pizza, C. Plant metabolites. New sesquiterpene and ionone glycosides from *Eriobotrya japonica*. *J. Nat. Prod*. **1992**, *55*, 1025–1032.
170. Lee, M.H.; Son, Y.K.; Han, Y.N. Tissue factor inhibitory sesquiterpene glycoside from *Eriobotrya japonica.* Arch. Pharm. Res. **2004**, *27*, 619–623.
171. Ao, X.; Zhao, L.; Li, H.; Ren, B.; Wu, H.; Chen, J.; Li, W. New sesquiterpene glycosides from the leaves of *Eriobotrya japonica. Nat. Prod. Commun*. **2015**, *10*, 1145–1147.
172. Zhao, L.; Chen, J.; Lv, H.; Ao, X.; Ren, B.; Li, W. A new sesquiterpene glycoside from the leaves of *Eriobotrya japonica. Chem. Nat. Compd.* **2015**, *51*, 1103–1106.
173. Chen, J.; Li,W.; Wu, J.; Ren, B.; Zhang, H. Hypoglycemic effects of a sesquiterpene glycoside isolated from leaves of loquat (*Eriobotrya japonica* (Thunb.) Lindl.). *Phytomedicine* **2008**, 15, 98–102.
174. Chen, J.; Li, W. Progress in studies on phytochemistry and biological activity of Folium Eriobotryae. *Med. Aromat. Plant Sci. Biotechnol*. **2008**, *2*, 18–23.
175. Tai, Q.; Xu, X.; Guo, W. Analysis of chemical composition of essential oil in leaves of *Eriobotrya japonica* by GC-MS. *Chinese Journal of Hospital Pharmacy* **2008**, 28, 206–208. (In Chinese)
176. Louati, S.; Simmonds, M.S.J.; Grayer, R.J.; Kite, G.C.; Damak, M. Flavonoids from *Eriobotrya japonica* (Rosaceae) growing in Tunisia. *Biochem. Syst. Ecol.* **2003**, *31*, 99–101.
177. Soung, D.Y.; Kim, J.S.; Chung, H.Y.; Jung, H.A.; Park, J.C.; Choi, J.S. Flavonoids and chlorogenic acid from *Eriobotrya japonica* scavenge peroxynitrite. *Nat. Prod. Sci*. **1999**, *5*, 80–84.
178. Ito, H.; Kobayashi, E.; Takamatsu, Y.; Li, S.H.; Hatano, T.; Sakagami, H.; Kusama, K.; Satoh, K.; Sugita, D.; Shimura, S.; Itoh, Y.; Yoshida, T. Polyphenols from *Eriobotrya japonica* and their cytotoxicity against human oral tumor cell lines. *Chem. Pharm. Bull.* **2000**, *48*, 687–693.
179. Ito, H.; Kobayashi, E.; Li, S.; Hatano, T.; Sugita, D.; Kubo, N.; Shimura, S.; Itoh, Y.; Tokuuda, H.; Nishino, H.; Yoshida, T. Antitumor activity of compounds isolated from leaves of *Eriobotrya japonica. J. Agr. Food Chem.* **2002**, *50*, 2400–2403.
180. Kawahara, N.; Satake, M.; Goda, Y. A new acylated flavonol glycoside from the leaves of *Eriobotrya japonica. Chem. Pharm. Bull.* **2002**, *50*, 1619–1620.
181. Pfisterer, P.H.; Shen, C.; Nikolovska-Coleska, Z.; Schyschka, L.; Schuster, D.; Rudy, A.; Wolber, G.; Vollmar, A.M.; Rollinger, J.M.; Stuppner, H. In silico discovery of acylated flavonol monorhamnosides from *Eriobotrya japonica* as natural, small-molecular weight inhibitors of XIAP BIR3. *Bioorganic. Med. Chem*. **2011**, *19*, 1002–1009.
182. Liu, Y.; Zhang, W.; Xu, C.; Li, X. Biological activities of extracts from loquat (*Eriobotrya japonica* Lindl.): a review. *Int. J. Mol. Sci*. **2016**, *17*.

183. Wu, H.; Cao, C.; Zhou, C. Determination of amygdalin in the fruit of *Eriobotrya japonica* Lindl by high performance liquid chromatography. *Biomed. Res.* **2017**, *28*, 8827–8831.
184. Cheng, L.; Liu, Y.; Chen, L.; Luo, J. Studies on the triterpenoidal saponins from flowers of *Eribotrya japonica*. *Hua Xi Yi Ke Da Xue Xue Bao.* **2001**, *32*, 283–285. (In Chinese)
185. Zhou, C.; Chen, K.; Sun, C.; Chen, Q.; Zhang, W.; Li, X. Determination of oleanolic acid, ursolic acid and amygdalin in the flower of *Eriobotrya japonica* Lindl. by HPLC. *Biomed. Chromatogr*. **2007**, *21*, 755–761.
186. Li, Q. Studies on components from the flowers of *Eiobotrya japonica* L. and its biological activity. M.D. Thesis, Sichuan Normal University, Sichuan, China, April 2009. (In Chinese)
187. Merle, H.; Blázquez, M.A.; Boira, H. Chemical composition of the essential oil of *Eriobotrya japonica* (Thunb.) Lindl. flowers in the western Mediterranean area. *CIHEAM (Options Méditerranéennes: Série A. Séminaires Méditerranéens)* **2003**. *58*, 191–193.
188. Esmaeili, A.H.; Moghaddam, A.H.; Chaichi, M.J. Identification, determination, and study of antioxidative activities of hesperetin and gallic acid in hydro-alcoholic extract from flowers of *Eriobotrya japonica* (Lindl.). *Avicenna J. Phytomed*. **2014**, *4*, 260–266.
189. Shen, T.; Wu, Z. Determination of amygdalin in the flowers of *Eriobotrya japonica* by HPLC. *Chinese Traditional and Herbal Drugs* **2012**, *43*, 2438–2439. (In Chinese)
190. Li, S. Reasearch on the aromatic components and essential oil from flowers of *Eriobotrya japonica*. M.D. Thesis, Fujian Agriculture and Forestry University, Fujian, China, April 2012. (In Chinese)
191. Chen, F.; Liu, X.; Lin, H.; Chen, L. Determination of organic acids from the fruit and leaf of loquat by ion-exchange chromatography. *Journal of Fujian Agriculture and Forestry University* **2004**, *33*, 195–199.
192. Fouedjou, R.T.; Nguelefack-Mbuyo, E.P.; Ponou, B.K.; Nguelefack, T.B.; Barboni, L.; Tapondjou, L.A. Antioxidant activities and chemical constituents of extracts from *Cordyline fruticosa* (L.) A. Chev. (Agavaceae) and *Eriobotrya japonica* (Thunb) Lindl, (Rosaceae). *Pharmacologia* **2016**, *7*, 103–113.
193. Yokota, J.; Takuma, D.; Hamada, A.; Onogawa, M.; Yoshioka, S.; Kusunose, M.; Miyamura, M.; Kyotani, S.; Nishioka, Y. Scavenging of reactive oxygen species by *Eriobotrya japonica* seed extract. *Biol. Pharm. Bull.* **2006**, *29*, 467–471.
194. Barbi, R.C.T.; Teixeira, G.L.; Hornung, P.S.; Avila, S.; Hoffmann-Ribani, R. *Eriobotrya japonica* seed as a new source of starch: assessment of phenolic compounds, antioxidant activity, thermal, rheological and morphological properties. *Food Hydrocoll.* **2018**, *77*, 646–658.
195. Jeong, J.; Lee, K.; Kim, S. Simultaneous determination of benzoic acid, caffeic acid and chlorogenic acid in seeds of *Eriobotrya japonica* and their antibacterial effect. *Journal of Applied Biological Chemistry* **2014**, *57*, 89–93.
196. Zhou, C.; Li, X.; Zhang, W.; Sun, C.; Chen, K. Oleanolic and ursolic acid in the fruit of *Eriobotrya japonica* Lindl. *J. Med. Plants Res.* **2011**, *5*, 1735–1740.
197. Agrawal, S.; Misra, K. Loquatoside - a new leucocyanin from *Eriobotrya japonica* fruits. *Planta Med*. **1980,** *38*, 277–278.
198. Hamauzu, Y.; Chachin, K.; Ding, C.; Kurooka, H. Differences in surface color, flesh firmness, physiological activity, and some components of loquat fruits picked at various stages of maturity. *J. JPN. SOC. HORTIC SCI.* **1997**, *65*, 859–865.
199. Ding, C.; Chachin, K.; Ueda, Y.; Imahori, Y.; Wang, C. Metabolism of phenolic compounds during loquat fruit development. *J. Agri. Food Chem.* **2001**, *49*, 2883–2888.
200. Sadana, J.C. Carotenoids of loquat (*Eriobotrya japonica* Lindl.). *Biochem. J*. **1949**, *44*, 401–402.
201. Fröhlich, O.; Schreier, P. Volatile constituents of loquat (*Eriobotrya japonica* Lindl.) fruit. *J. Food Sci*. **1990**, *55*, 176–180.
